# Two-Pore-Domain Potassium (K_2P_-) Channels: Cardiac Expression Patterns and Disease-Specific Remodelling Processes

**DOI:** 10.3390/cells10112914

**Published:** 2021-10-27

**Authors:** Felix Wiedmann, Norbert Frey, Constanze Schmidt

**Affiliations:** 1 Department of Cardiology, University of Heidelberg, 69120 Heidelberg, Germany; Felix.Wiedmann@med.uni-heidelberg.de (F.W.); norbert.frey@med.uni-heidelberg.de (N.F.); 2 DZHK (German Center for Cardiovascular Research), Partner Site Heidelberg/Mannheim, University of Heidelberg, 69120 Heidelberg, Germany; 3 HCR, Heidelberg Center for Heart Rhythm Disorders, University of Heidelberg, 69120 Heidelberg, Germany

**Keywords:** K_2P_-channel, TASK-1, TREK-1, two-pore-domain potassium channel

## Abstract

Two-pore-domain potassium (K_2P_-) channels conduct outward K^+^ currents that maintain the resting membrane potential and modulate action potential repolarization. Members of the K_2P_ channel family are widely expressed among different human cell types and organs where they were shown to regulate important physiological processes. Their functional activity is controlled by a broad variety of different stimuli, like pH level, temperature, and mechanical stress but also by the presence of lipids or pharmacological agents. In patients suffering from cardiovascular diseases, alterations in K_2P_-channel expression and function have been observed, suggesting functional significance and a potential therapeutic role of these ion channels. For example, upregulation of atrial specific K_2P_3.1 (TASK-1) currents in atrial fibrillation (AF) patients was shown to contribute to atrial action potential duration shortening, a key feature of AF-associated atrial electrical remodelling. Therefore, targeting K_2P_3.1 (TASK-1) channels might constitute an intriguing strategy for AF treatment. Further, mechanoactive K_2P_2.1 (TREK-1) currents have been implicated in the development of cardiac hypertrophy, cardiac fibrosis and heart failure. Cardiovascular expression of other K_2P_ channels has been described, functional evidence in cardiac tissue however remains sparse. In the present review, expression, function, and regulation of cardiovascular K_2P_ channels are summarized and compared among different species. Remodelling patterns, observed in disease models are discussed and compared to findings from clinical patients to assess the therapeutic potential of K_2P_ channels.

## 1. Introduction

**Figure 1:**
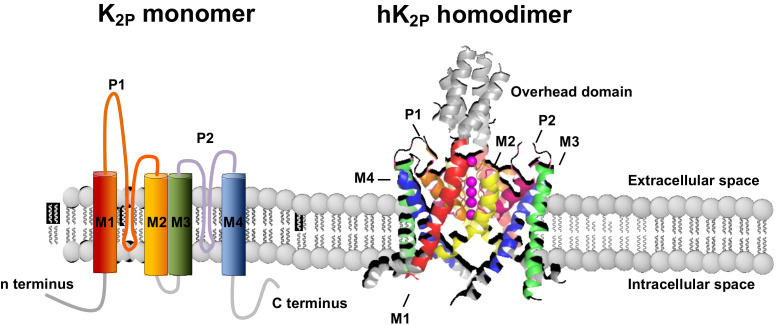
Membrane topology and structure of K_2P_ channels. K_2P_ channel monomers (**left**), consisting of 4 transmembrane domains (M1–4) and 2 pore forming loops (P1–2) assemble as homo- or heterodimers. (**right**).

Two-pore-domain potassium (K_2P_) channels are expressed throughout the human body and contribute to background potassium conductance in many different cell types [1,2]. In the human genome 15 K_2P_ channels have been described which differ from classical potassium channels by the fact that each subunit carries two pore-forming domains, and the channels thus assemble as dimers instead of tetramers (Figure 1). K_2P_ channels give rise to background or “leak” potassium currents which control a multitude of physiological processes [1]. Initially, K_2P_ currents were described as outward rectifying “leakage currents” but recent work has shown that several members of the K_2P_ family can also be voltage activated [3].

**Figure 2:**
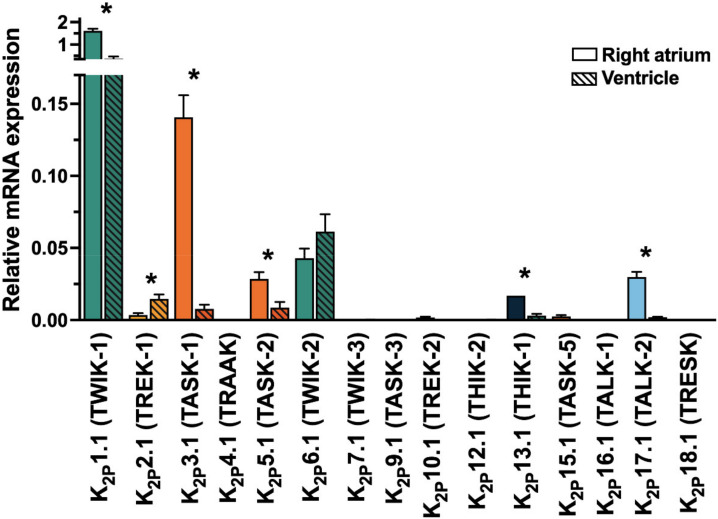
Cardiac mRNA levels of K_2P_ channels in the human heart (whole tissue). Expression of two-pore-domain potassium (K_2P_-) channel mRNA level in human right atrial (*n* = 10) and left ventricular (*n* = 5) tissue samples. Data are given as mean ± SEM relative to the housekeeping gene importin 8 (*IPO8*). * indicate *p* < 0.05 from Student’s t-tests. Data from Schmidt et al. 2015, Circulation [8].

**Figure 3:**
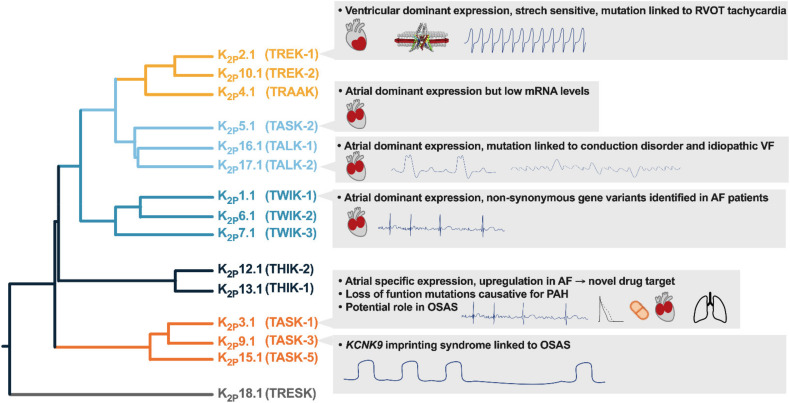
Potential translational implications of cardiac K2P channel expression.AF, atrial fibrillation; OSAS, obstructive sleep apnea; PAH pulmonary arterial hypertension; RVOT, right ventricular outflow tract; VF, ventricular fibrillation.

K_2P_ currents show a high degree of similarity to the potassium plateau currents *I_KP_*, described in guinea-pig cardiomyocytes and the steady-state potassium current *I_K,SS,_* characterized in murine cardiomyocytes and the arachidonic acid-sensitive potassium current *I*_KAA_ from rat ventricular cardiomyocytes [4,5,6,7]. Cardiac mRNA abundance was described for several members of the K_2P_ family (Figure 2) In the present review, expression, function, and regulation of cardiovascular K_2P_ channels are summarized and compared among different species. Remodelling patterns, observed in disease models are discussed and compared to findings from clinical patients to assess the therapeutic potential of K_2P_ channels (Figure 3).

## 2. Structural Assembly and Nomenclature of K_2P_ Channels

The 15 channel subunits of the K_2P_ family each consists of around 300 and 550 amino acids. The sequence differences between the individual subunits of the K_2P_ channel can sometimes be as large as to other potassium channel families. K_2P_18.1 (TRESK) channel subunits, for example share only 19% amino acid sequence identity with the other K_2P_ family members. But the common feature that links them is the eponymous structural motif of two pore-forming domains per subunit, which distinguishes them from all other potassium channel groups. As shown in Figure 1, the four alphahelical transmembrane domains (M1–M4) flank two pore-forming loops (P1 and P2), each containing the potassium selective filter motif (GLG, GFG, or GYG). M1 and P1 are connected by a long extracellular loop, forming an overhead cap structure. The short amino terminus and a much longer carboxy terminus, which contains a variety of regulatory phosphorylation and protein interaction motifs, are localized intracellularly. Whereas most potassium channels form tetramers with one pore-forming loop per subunit, a functional two-pore domain potassium channel is composed of two alpha subunits (Figure 1). In addition to homodimerization, certain K_2P_ channel subunits can also assemble as heterodimers. This is mainly described within the same subfamilies (i.e., TASK-1/TASK-3, TREK-1/TRAAK, THIK-1/THIK-2), but can also occur between TWIK-1 and TREK or TASK-1, and between TASK-1/TALK-2 subunits. Physiological relevance in the perception of hypoxia has been described for TASK-1/TASK-3 heterodimers and TWIK-1/TREK-1 heterodimers have been detected in astrocytes. Apart from the TASK-1 and TALK-1 subfamilies, all K_2P_ channel subunits possess a conserved Cys-amino acid residue of the overhead domain that is thought to play a major, although not yet conclusively elucidated, role in dimerization. The predicted membrane topology and tertiary structure have already been confirmed by X-ray structural analysis for several K_2P_-channels (Table 1).

Upon their discovery, the individual K_2P_-channels received trivial names reflecting their respective structural and regulatory properties: TWIK: “Tandem of P domains in a weak inward rectifying K^+^ channel”, TREK: “TWIK-related K^+^ channel”, TASK: “TWIK-related acid-sensitive K^+^ channel”, TRAAK: “TWIK-related arachidonic acid activated K^+^ channel”, TALK “TWIK-related alkaline pH-activated K^+^ channel”, THIK “tandem pore domain halothane-inhibited K^+^ channel”, and TRESK “TWIK-related spinal cord K^+^ channel”. In parallel, the channels are labeled consecutively with the designations K_2P_1.1 to K_2P_18.1 according to the Human Genome Organization name of the encoding gene (*KCNK1* to *KCNK18*) (see Figure 2 and Table 1). Each of the 15 subfamilies members (K_2P_1.1 to K_2P_18.1) contains only one member. Unfortunately, this led to a confusing nomenclature in which channels with different functional properties such as TASK-1 and TASK-2 have similar names, while other channels are titled with acronyms of factually incorrect names (for example, TWIK-1 is not a weak inward rectifier but an open rectifier). Further, some channels carry a variety of redundant names such as in case of K_2P_3.1: TBAK1, TASK1 and OAT1. Several *KCNKx* designators were initially assigned to K_2P_ channel transcripts that later turned out to be orthologs of other human K_2P_ channels. Thus, *KCNK8* (the murine transcript designated *kcnk8* later proved to be an ortholog of human *KCNK7* and was therefore renamed *kcnk7*), *KCNK11*, and *KCNK14* (both orthologs of *KCNK15*) do not exist [9]. For better understanding, we will provide the trivial names of the channel subunits in brackets in addition to the International Union of Basic and Clinical Pharmacology IUPHAR (K_2P_X.1) names. Since they do not show any functional activity in heterologous expression systems, the channels *KCNK7*, *KCNK12* and *KCNK15* are referred to as silent K_2P_ channels. It remains unclear whether these K_2P_ channel subunits are truly nonfunctional in vivo or whether they just lack essential cofactors to achieve functionality upon heterologous expression. In fact, the functionality of the K_2P_16.1 channels could be restored by deletion of an n-terminal ER-retention motif [8].

## 3. K_2P_1.1 (TWIK-1)

Robust cardiac mRNA levels were consistently described for *KCNK1* [10,11,12,13,14,15]. In a study from our laboratory, which examined the expression of all K_2P_ channels in the human heart (TaqMan-qPCR; Figure 2), the highest mRNA levels were detected for *KCNK1* [10]. Atrial predominant mRNA abundance was shown in patient-derived tissue samples but not in rodents (Table 2) [10,16].

The zebrafish possess two orthologues of the human *KCNK1* gene, *kcnk1a* and *kcnk1b* which, most likely as the result of an ancient genome duplication, both encode functional TWIK-1 channels. Knockdown of *kcnk1a* or *kcnk1b* in zebrafish embryos resulted in a phenotype atrial dilatation and bradycardia, suggesting a role of K_2P_1.1 (TWIK-1) in regulation of sinus node function and structural heart development [11]. Further, downregulation of cardiac *Kcnk1* mRNA levels was reported in a diabetic rat model, displaying again a phenotype of sinus bradycardia [19]. The presence of single nucleotide polymorphisms in the *KCNK1* gene might be correlated with the prevalence of coronary artery disease [79]. Christensen et al. reported the identification of three non-synonymous *KCNK1* gene variants (p.R171H, p.I98M, and p.G236S) in a cohort of 373 atrial fibrillation (AF) patients. Although these variants are localized in highly conserved domains, no effect on potassium current, reversal potential, or subcellular localization was detected in heterologous expression systems [11]. Pharmacological modulation of homodimeric K_2P_1.1 (TWIK-1) channels by quinine and quinidine was described (Table 3) [20]. In our own studies, AF and heart failure patients showed unchanged cardiac *KCNK1* mRNA levels [10,40], while others reported upregulation of *KCNK1* mRNA patients with atrial dilatation [11] or Brugada syndrome [80], downregulation of *KCNK1* mRNA in AF [12] or mitral valve disease [81].

The physiological role of K_2P_1.1 (TWIK-1) channel subunits has not been conclusively clarified, mostly due to lack of specific inhibitors and its very low currents in heterologous expression systems [82]. If measurable, heterologously expressed K_2P_1.1 (TWIK-1) channel homodimers give rise to potassium currents that are sensitive to acidic pH as well as external K^+^ concentration [134]. Therefore, it was speculated whether K_2P_1.1 (TWIK-1) might contribute to cardiac *I*_K1_, *I*_KAch_, *I*_KATP_, and *I*_TASK_ currents [11,12,13,14,80,135,136]. Altered ion conductivity under low extracellular potassium concentrations (for example Na^+^ permeability, which shifts homodimeric K_2P_1.1 (TWIK-1) channels from an inhibitory to an excitatory channel) could link K_2P_1.1 (TWIK-1) channels to the pathophysiology of hypokalemia-induced rhythm disturbances [137]. Through its ability to heterodimerize with other K_2P_ subunits, K_2P_1.1 (TWIK-1) subunits could modulate the pharmacological and functional properties of atrial K_2P_3.1 (TASK-1) channel subunits [138,139,140].

## 4. K_2P_2.1 (TREK-1)

Mechanosensitivity is a unique feature of the TREK/TRAAK subfamily, as these K_2P_ channels are activated by membrane stretch and osmotic swelling [141]. Temperature, lipids, extracellular or intracellular pH, anesthetics or other drugs, phosphorylation, glycosylation, G protein-coupled receptors and other protein partners represent further regulators of homodimeric K_2P_2.1 (TREK-1) channels [97,124,141,142,143,144]. The versatility of this channel is further enhanced by alternative translation initiation (ATI) variants that differ in spatiotemporal expression, single-channel conduction, ion selectivity and regarding their pharmacological profile [43,145,146]. Further, K_2P_2.1 (TREK-1) channel subunits are reported to from heterodimers with K_2P_1.1 (TWIK-1), K_2P_4.1 (TRAAK) and K_2P_10.1 (TREK-2) [147,148].

In the rat heart, *Kcnk2* mRNA and protein expression has been described in both atrial and ventricular tissue samples (Table 2) [28,29,32,33,149]. However, in the mouse heart, most studies describe ventricular-dominant K_2P_2.1 (TREK-1) expression or mRNA abundance patterns [16,26,41]. Abundant K_2P_2.1 (TREK-1) expression was also detected in the porcine heart, with the highest expression levels in the sinoatrial and atrioventricular nodal tissue [36,37] and in human cardiac tissue samples, where again ventricular dominant K_2P_2.1 (TREK-1) expression could be observed [10,37,40,41]. Interestingly, a transmural gradient of ventricular K_2P_2.1 (TREK-1) expression levels was described with endocardial expression levels 17-fold higher than that in the epicardium, [30,149]. Strikingly, this gradient seems to parallel transmural changes in stretch-activated potassium currents, as mechanical stretch has been shown to cause increased action potential shortening in subendocardial cardiomyocytes compared to the subepicardium [150]. In a similar fashion chloroform-activated K_2P_2.1 (TREK-1)-like currents are significantly larger in endocardial than epicardial cells [30].

Homodimeric K_2P_2.1 (TREK-1) channels are inhibited by the anticonvulsant drugs valproate, gabapentin and carbamazepine [102] by the antidepressants like fluoxetine, paroxetine, citalopram or escitalopram (Table 3) [96,102], and the antipsychotics haloperidol or clozapine [101]. While some of these interactions would only be relevant at supratherapeutic plasma levels, others already have an impact in the physiological range [141]. It has therefore been speculated whether the blockade of cardiac K_2P_2.1 (TREK-1) channels could contribute to the proarrhythmic potential of these compounds [41,141]. Remarkably, K_2P_2.1 (TREK-1) knockout was shown to cause a phenotype of QT interval prolongation, linking loss of cardiac K_2P_2.1 (TREK-1) to QT prolongation [151]. Likewise, antiarrhythmic drugs were described to block K_2P_2.1 (TREK-1) channels: Vaughan Williams class I compounds lidocaine, mexiletine and propafenone, class III antiarrhythmic drugs dronedarone and vernakalant, the beta-blocker carvedilol and late sodium current inhibitor ranolazine were identified as in vitro K_2P_2.1 (TREK-1) inhibitors (Table 3) [43,82,84,104,106,109]. Since IC_50_ levels are mostly in the supratherapeutic range, it is unclear to what extent inhibition of K_2P_2.1 (TREK-1) contributes to the antiarrhythmic effects of these compounds.

In isolated rat ventricular cardiomyocytes the mechano-, pH-, and arachidonic acid-sensitive potassium current *I*_KAA_ displays a number of further features like activation by volatile anesthetics, inhibition by cAMP analogues as well as beta-adrenergic receptor agonists, the absence of a relevant voltage dependency, a specific single-channel conductance and burst mode activity, which identify it as a K_2P_2.1 (TREK-1) current (Table 4) [7,28,29,32,33,149,152]. Further, resting membrane potentials of chicken embryo-derived atrial cardiomyocytes are regulated by K_2P_2.1 (TREK-1) [153]. Finally, cardiomyocyte-specific K_2P_2.1 (TREK-1) knockout mice exhibit a phenotype of stress-induced sick sinus syndrome and prolongation of QT intervals that could be reproduced in a transgenic model which employed C-terminal truncation of beta IV spectrin to disrupt its interaction with K_2P_2.1 (TREK-1), thereby impairing intracellular K_2P_2.1 (TREK-1) protein trafficking [27,151]. In a similar fashion, knockout of K_2P_2.1 (TREK-1) channel surface targeting by its protein partners POPDC1 or POPDC2 revealed a phenotype of exercise-induced and age-dependent sick sinus syndrome [154,155], while a double-knockout mouse displayed AV conduction disturbance [156]. Moreover, a familial autosomal recessive POPDC1 mutation has been associated with the phenotype of limb-girdle muscular dystrophy type X2 in combination with AV block [157] and POPDC2 mutations have been shown to cause AV block without a skeletal muscle phenotype [158].The fact that K_2P_2.1 (TREK-1) channels are activated in acidosis and by mechanical stress has given rise to speculation about a role of this channel in the development of cardiac arrhythmias for more than two decades [28]. Metabolic changes associated with myocardial ischemia lead to a decrease in pH. By activating K_2P_2.1 (TREK-1), this can cause a dispersion of repolarization and consecutively the development of arrhythmias. Similarly, altered wall tension due to hypertension, valvular vitiation, in the margins of myocardial scars, or AF may activate K_2P_2.1 (TREK-1) [141,158,159]. Recently, a heterozygous missense mutation (I267T) of K_2P_2.1 (TREK-1) was identified in a patient with idiopathic right ventricular outflow tract tachycardia [160]. This mutation results in an amino acid exchange from isoleucine to threonine in close proximity to the selectivity filter of the channel, leading to increased stretch sensitivity and sodium permeability.

In a murine model of transverse aortic constriction (TAC)-induced pressure overload upregulation of ventricular *Kcnk2* mRNA expression was described [16]. In a similar fashion, K_2P_2.1 (TREK-1) protein levels were increased in a rat model of isoproterenol-induced left ventricular hypertrophy [149]. Global K_2P_2.1 (TREK-1) knockout mice showed an exaggerated form of pressure overload-induced concentric ventricular hypertrophy, which could be prohibited only by fibroblast-specific deletion of K_2P_2.1, (TREK-1) whereas the cardiomyocyte-specific knockout of K_2P_2.1 (TREK-1) resulted in cardiac dysfunction under pressure-overload conditions [161]. In a murine atrial fibrillation (AF) model of CREM-IbΔC-X transgenic mice, downregulation of atrial K_2P_2.1 (TREK-1) mRNA and protein levels were observed [16,41]. It, however, remains uncertain whether this is also the case for AF patients: while one study described AF-associated downregulation of atrial K_2P_2.1 (TREK-1) [37] others merely describe a trend that does not reach statistical significance [10,40,41]. One possible explanation is the remote regulation of atrial K_2P_2.1 (TREK-1) expression by ventricular heart failure, a mechanism recently described for K_2P_3.1 (TASK-1) [40] and also observed for K_2P_2.1 (TREK-1) in another study [41]. Indeed, in contrast to the other study, the cohort of patients characterized in the former study was performed in patients who all suffered from severe heart failure. In a similar fashion, a strong trend towards downregulation of atrial *Kcnk2* mRNA could be observed in a murine model of TAC-induced pressure overload [16]. Furthermore, downregulation of atrial K_2P_2.1 (TREK-1) protein expression was described in a porcine model of combined AF and heart failure [36] and gene therapeutic restoraton of K_2P_2.1 (TREK-1) expression was able to attenuate the AF phenotype [37].

For a more detailed description of the cardiac role of K_2P_2.1 (TREK-1), we would like to refer to the following literature [41,141,158].

## 5. K_2P_3.1 (TASK-1)

Among the entire K_2P_ family, K_2P_3.1 (TASK-1) is the channel with the best characterized cardiac significance. K_2P_3.1 (TASK-1) channels are expressed in neuronal tissue, cardiomyocytes, vascular smooth muscle cells, the carotid body glomus, the adrenal gland, brown adipose tissue and immunocytes, where they control important physiological processes [2,115]. K_2P_3.1 (TASK-1) channels are regulated by a number of different stimuli, such as pH level, hypoxia, PKA, PKC, or PLC activity, and several drugs like volatile anesthetics [2].

In the murine and the rat heart, *KCNK3* mRNA was detected, both in atrial as well as in ventricular tissue samples (Northern blot, RT-PCR, Taq-Man qPCR; Table 2) [15,16,18,25,26,34,44,45,47]. Humans, however, show an almost atrial-specific K_2P_3.1 (TASK-1) expression within the heart with 14- to 16-fold lower expression levels in ventricular tissue (RT-PCR, Taq-Man qPCR, microarray, bulk RNAseq, Western blot) [10,12,14,39,40,49,54,56,57]. In guinea pigs and domestic swine, atrial-specific K_2P_3.1 (TASK-1) expression was also described [49,51,52,53,54].

Several clinically relevant antiarrhythmic drugs have been identified to inhibit homodimeric K_2P_3.1 (TASK-1) channels at either physiological or subtherapeutic concentrations (Table 3). Among them are the class I antiarrhythmic drugs propafenone, mexiletine, lidocaine, and quinidine [104,122,123], the betablockers propranolol and carvedilol [42], class III antiarrhythmics amiodarone and dronedarone [82,110] as well as cardiac glycosides [111] and ranolazine [109]. The respiratory stimulant doxapram was further identified as a potent blocker of both K_2P_3.1 (TASK-1) and K_2P_9.1 (TASK-3) channels through which it presumably exerts the main part of its respiratory drive-increasing effect [119,175]. Furthermore, preclinical experimental antiarrhythmic drugs developed as specific inhibitors of the K_V_1.5 channel (A239 [AVE1231], A1899 [S20591], AVE0118, S9947, MSD-D, and ICAGEN-4) are potent K_2P_3.1 (TASK-1) inhibitors [117]. Although no direct structural similarities of the pore regions of both channels could be detected, these compounds were shown to be 1.4- to 70-fold more potent K_2P_3.1 (TASK-1) inhibitors as compared to K_V_1.5 [117]. In addition, bisamides represent a new class of high-affinity K_2P_3.1 (TASK-1) inhibitors with IC_50_ values in the single-digit nanomolar range, as in the case of compound ML365 (Table 3) [116].

Availability of high-affinity inhibitors enables functional detection of K_2P_3.1 (TASK-1) currents in isolated cardiomyocytes. K_2P_3.1 (TASK-1) currents were isolated from rat ventricular cardiomyocytes by lowering pH, activation of cardiac α1-adrenergic receptors and by administration of the inhibitor A293 (Table 4) [15,162,163]. Patch-clamp measurements of murine K_2P_3.1 (TASK-1) current could be confirmed by the use of *Kcnk3* knockout mice [25] and likewise, functional detection of K_2P_3.1 (TASK-1) currents was achieved by patch-clamp technique in isolated porcine [52,53,54,164] and human atrial cardiomyocytes, where a significant APD prolongation could be demonstrated [10,39,40,53,56]. Under physiological conditions, *I*_TASK-1_ was identified to carry up to 28% of the background potassium current in isolated human atrial cardiomyocytes [39].

In induced pluripotent stem cell- (iPS-) derived cardiomyocytes (iPSC), APD values could be prolonged by transfection of K_2P_3.1 (TASK-1) siRNA [22]. In a zebrafish model, a decreased heart rate was observed after K_2P_3.1 (TASK-1) knockdown, which was accompanied by an increased atrial diameter [165]. In excised guinea pig hearts, APD remained unchanged upon K_2P_3.1 (TASK-1) inhibition with A293 or ML365. Switching the pH level from pH 7.4 to 7.8, however, resulted in significant prolongation of atrial effective refractory periods [49]. Global *Kcnk3* knockout mice exhibited a phenotype of QTc prolongation (around 30%), prolongation of single cell APDs or monophasic action potentials and a broad QRS complex [25,26]. In transgenic *Kcnk3* knockout rats, APD prolongation as well as resting membrane depolarization was described [163].

In a porcine large animal model of AF, atrial K_2P_3.1 (TASK-1) expression was found to be significantly upregulated (TaqMan qPCR, western blot, patch-clamp electrophysiology) [52,141,164]. These results could also be reproduced on atrial tissue samples from atrial fibrillation patients (TaqMan qPCR, microarray, bulk RNAseq, western blot, patch-clamp electrophysiology) [10,41,55,57]. Considering its atrial-specific expression, its effect on atrial APD, and its upregulation in patients with AF, K_2P_3.1 (TASK-1) channels combine several properties that make it an ideal molecular target for the treatment of AF.

Inhibition of K_2P_3.1 (TASK-1) in cardiomyocytes from AF patients has been shown to counteract AF-induced APD shortening [104,154]. After administration of A293 (200 nM), APDs of atrial cardiomyocytes isolated from AF patients could be prolonged around 30% to values observed in sinus rhythm controls [104,154]. After intravenous application of K_2P_3.1 (TASK-1) inhibitors in healthy control pigs, significant prolongation of both, atrial effective refractory periods and ADP values pointed towards class III antiarrhythmic effects of K_2P_3.1 (TASK-1) inhibition [53,54]. Furthermore, the inducibility of atrial arrhythmias was significantly reduced by K_2P_3.1 (TASK-1) inhibitors in different studies [176,177,178]. In a similar fashion, intravenous administration of K_2P_3.1 (TASK-1) inhibitors A293 and doxapram led to rapid, safe and successful cardioversion of artificially induced AF episodes in a porcine large animal model [53,54]. These antiarrhythmic effects could further be employed for rhythm control in a porcine model of burst pacing induced “persistent” AF, induced via implanted pacemakers using a biofeedback algorithm [53,164] and reproduced with an AAV-mediated anti-K_2P_3.1 (TASK-1) gene therapy approach [52]. Based on these encouraging results, the currently ongoing DOCTOS trial (doxapram conversion to sinus rhythm; EudraCT No: 2018-002979-17) was started, which investigates whether the FDA and EMA approved K_2P_3.1 (TASK-1) inhibitor doxapram can cardiovert AF in patients [2,179].

Interestingly, also reduction of atrial K_2P_3.1 (TASK-1) expression was linked to AF as in a dog model of postoperative AF, a phosphorylation dependent downregulation of K_2P_3.1 (TASK-1) was reported [50] and CREM-TG AF mice display atrial downregulation of K_2P_3.1 (TASK-1) in conjunction with massive atrial dilatation and scarring [16]. Patients who suffer from reduced left ventricular ejection fraction display reduced atrial K_2P_3.1 (TASK-1) expression, independently from their rhythm state [40]. Finally, three genetic variants (two kozak variants and missense variant K_2P_3.1 (TASK-1) V123L mutation all of which reduce the expression or channel function) were found in patients with familial AF [49].

In addition to its role in the control of heart rhythm, K_2P_3.1 (TASK-1) is also discussed as a regulator of cardiac energetics and metabolic function, as *Kcnk3* knockout mice were protected from pressure overload-induced cardiomyopathy. Compared to wild-type littermates, *Kcnk3* knockout mice showed a preservation of systolic as well as diastolic function and a relative abrogation in concentric left ventricular hypertrophy upon TAC-induced pressure overload [46].

Moreover, K_2P_3.1 (TASK-1) channels were described to be expressed in in human pulmonary artery smooth muscle cells, where they serve as regulators of the basal membrane potential and consecutively regulate pulmonary vascular tone [180]. Furthermore, *KCNK3* loss-of-function mutations were found to cause idiopathic pulmonary arterial hypertension [166] and acute pharmacological K_2P_3.1 (TASK-1) inhibition in pigs led to a mild but significant increase in invasively measured pulmonary arterial pressure [164]. In the context of adrenal *KCNK3* expression, a role of the K_2P_3.1 (TASK-1) channel in aldosterone secretion and blood pressure control is further discussed. Global *Kcnk3* knockout mice display a phenotype of mild hyperaldosteronism [181] and single nucleotide polymorphisms in the *KCNK3* gene were associated with plasma aldosterone levels [182]. Accordingly, elevated systolic blood pressure values were described in the *Kcnk3* knockout mouse [25]. Finally, K_2P_3.1 (TASK-1) channels are also discussed to be involved in regulating function of immune cells and in thermogenesis in brown adipose tissue [183]. Thus, there is a need for further studies that exclude systemic side effects in the use of TASK-1 inhibitors for treatment of AF.

## 6. K_2P_4.1 (TRAAK)

Although it was suspected about 20 years ago, that the K_2P_4.1 (TRAAK) channel, based on northern blot analysis, might be mainly expressed in the human heart there is little evidence to date for a cardiac role of this K_2P_ channel. Several studies reported cardiac *KCNK4* mRNA expression, mostly with atrial predominant expression patterns (TaqMan qPCR; Table 2) in human as well as in murine heart tissue samples [10,22,26,41]. Compared with other cardiac ion channels, however, expression levels were relatively low [10,16,41]. A mild inhibitory effect of vernakalant and the late sodium channel blocker ranolazine has also been described for hK_2P_4.1 (TRAAK) homodimeric channels (Table 3) [83,109].

*Kcnk4* knockout mice were reported to display smaller ischemic areas upon cerebral infarction. No obvious phenotype of heart rhythm disorder or heart failure was described, and the mice were reported as viably and healthy [167,168]. We are, however, not aware of any studies that explicitly study the cardiac phenotype of these transgenic mice (Table 4).

## 7. K_2P_5.1 (TASK-2)

Shortly after the first description of the *KCNK5* gene, RT-PCR experiments had already indicated robust cardiac abundance of *KCNK5* mRNA [184], while other studies (RT-PCR) considered the cardiac mRNA levels to be rather low (Table 2) [22,23,26,38]. Our own studies indicated atrial predominant *KCNK5* mRNA abundance within the human and murine heart [10,16]. Further, a trend towards downregulation of atrial *KCNK5* mRNA in patients, suffering from chronic AF was noted that did not reach statistical significance [10]. K_2P_5.1 (TASK-2) homodimers are a molecular target on volatile and amide type local anesthetics (Table 3) [185,186] and inhibited by supratherapeutic concentrations of ranolazine [109]. siRNA transfection experiments pointed towards a functional role of K_2P_5.1 (TASK-2) in setting the membrane potential of pulmonary artery myocytes [187]. In the diabetic rat model with sinus bradycardia, mentioned above, downregulation of cardiac *Kcnk5* mRNA expression was reported (Table 4) [19]. Finally, genome-wide association studies could identify a risk locus, associated with the development of coronary artery disease and migraine within the *KCNK5* gene [188].

Breeding of global *Kcnk5* knockout mice resulted in a small number of female homozygous offspring, pointing towards a phenotype which might cause antenatal mortality [169]. Further, Gerstin et al. reported that one homozygote female animal was found dead in the cage at 12 days of age [169]. However, whether this was associated with cardiomyopathy or arrhythmia remains speculative.

## 8. K_2P_6.1 (TWIK-2)

Robust cardiac expressions patterns of *KCNK6* mRNA, derived from RT-PCR were described [10,18,22], while others report mild to moderate cardiac expression of this channel (RT-PCR, WB; Table 2) [15,23,26]. Interestingly, mRNA levels were reported to be significantly higher in the adult as compared to the neonatal rat heart [18]. Furthermore, abundant *Kcnk6* mRNA levels were found in rat saphenous arteries [189]. Upon TAC-induced pressure overload, an upregulation of murine ventricular *Kcnk6* mRNA could be observed (Table 4) [16]. *Kcnk6* deficient mice are hypertensive and display elevated RV pressure level as well as enhanced vascular contractility which was linked to enhanced rho kinase activity [170,171,172]. The physiological relevance of K_2P_6.1 (TWIK-2) is under debate because these channels conduct only low currents in the heterologous expression system [82]. It further was recently reported that K_2P_6.1 (TWIK-2) channel subunits give rise to functional K_2P_ currents in endolysosomes, where they affect the size and number of lysosomes [190] so it remains unclear whether the cell membrane is indeed the actual site of action of these channels.

## 9. K_2P_7.1 (TWIK-3)

The mainly neuronally detected K_2P_7.1 (TWIK-3) channel is a silent K_2P_ channel without proven potassium conductance in heterologous expression systems [191]. Only very low cardiac expression levels have been described for *KCNK7* (RT-PCR, TaqMan qPCR; Table 2) [10,23]. It was, however speculated whether its mRNA expression might be upregulated in atrial tissue samples, derived from AF patients [63]. Although not explicitly cardiac characterized, a global *Kcnk7* knockout mouse showed no obvious cardiac phenotype. Homozygous transgenic mice and wild-type littermates did not differ significantly in general appearance, gross anatomy, locomotion, or overt behavior (Table 4) [173].

## 10. K_2P_9.1 (TASK-3)

The cardiac relevance of K_2P_9.1 (TASK-3) channel subunits which are primarily known for their role in apoptosis, aldosterone secretion and tumor genesis remains controversial. Whereas most studies detected only relatively low mRNA levels in the human heart (qPCR, TaqMan qPCR; Table 2) [10,22,26,49], others showed high atrial expression, almost comparable to K_2P_3.1 (TASK-1) (RT qPCR, IF) [56]. In the rodent heart, low *Kcnk9* (TASK-3) mRNA abundance been described [15,16,18,25,26,48].

Echocardiographic characterization of *Kcnk9* knockout mice revealed a phenotype of concentric left ventricular hypertrophy with preserved ejection fraction (Table 4) [46]. In contrast to *Kcnk3* knockout mice, however, these animals are not TAC resistant, and heart failure symptoms are more likely to occur at a later time point [46]. Downregulation of ventricular *KCNK9* mRNA expression (TaqMan qPCR) in heart failure patients might point towards a pathophysiological role of this channel [22].

Single channel patch-clamp measurements, performed in isolated human atrial cardiomyocytes were able to detect a channel with characteristics corresponding to a heteromer of K_2P_3.1 (TASK-1) and K_2P_9.1 (TASK-3) [56]. However, besides this heteromeric and homodimeric K_2P_3.1 (TASK-1) channels, no current corresponding to a homodimeric K_2P_9.1 (TASK-3) channels could be detected. Functional studies in motoneurons or in rat carotid body glomus cells indicate that the K_2P_3.1 (TASK-1)/ K_2P_9.1 (TASK-3) heterodimer portion was about 52–75% and thus only a minority of K_2P_3.1 (TASK-1) channels are expressed as monomer at the cell surface [192,193]. Since the pharmacological properties of homodimeric and heterodimeric channels differ, heterodimerization has to be taken into account when targeting the K_2P_3.1 (TASK-1) channel in the treatment of cardiac arrhythmias.

A rare genetic disease, *KCNK9* imprinting syndrome, also known as Birk-Barel Syndrome is inherited in an autosomal dominant, maternally imprinted manner and associated with congenital central hypotonia, severe feeding difficulties, delayed development, and dysmorphic manifestations [194]. While no direct cardiac manifestation has been described to date, affected individuals may develop obstructive sleep apnea syndrome, which is particularly interesting because it again links the K_2P_ channels of the TASK subfamily to this disease entity.

Together with K_2P_3.1 (TASK-1), K_2P_9.1 (TASK-3) contributes to peripheral and central respiratory regulation [195]. Therefore, these K_2P_-channels are likely to constitute a molecular target of the respiratory stimulant doxapram [53]. K_2P_9.1 (TASK-3) homodimers are further inhibited by the class III antiarrhythmic drug dronedarone [82] and the antianginal drug ranolazine [109].

Hopefully, the recently available high-affinity K_2P_9.1 (TASK-3) inhibitors and activators will help to answer the question of the functional relevance of K_2P_9.1 (TASK-3) channels in cardiomyocytes.

## 11. K_2P_10.1 (TREK-2)

The role of K_2P_10.1 (TREK-2) channel subunits has so far been characterized mainly in the central nervous system (CNS), where this channel shows ubiquitous expression. However, a *KCNK10* knockout mouse showed remarkably few neurobehavioral phenotypes besides discrete abnormalities in anxiety-related behavior [174]. A cardiac phenotype of this mouse has not been described yet. Pharmacological in vitro measurements revealed vernakalant and carvedilol as inhibitors of K_2P_10.1 (TREK-2) homodimer channels (Table 3) [43,83]. Low cardiac mRNA abundance was described by our group and others (RT-PCR, TaqMan qPCR; Table 2) [10,15,22,40]. However, the expression patterns appeared atrial-predominant both in murine and patient-derived samples [10,41]. No relevant changes of K_2P_10.1 (TREK-2) expression could be detected in murine disease models of TAC-induced pressure overload or CREM-TG AF (Table 4) [16]. However, in right and left atrial patient-derived tissue samples, significant mRNA upregulation was demonstrated upon systolic heart failure [41].

## 12. K_2P_12.1 (THIK-2)

K_2P_12.1 (THIK-2) is referred to as a silent K_2P_-channel. This is likely due to both, a N-terminal retention signal and a low endogenous open probability [196]. While cardiac K_2P_12.1 (THIK-2) mRNA levels (RT-PCR, TaqMan qPCR) were described to be rather low (Table 2) [10,15,16,67], K_2P_12.1 (THIK-2) expression was detected in rat saphenous arteries [189] and might therefore be of relevance in control of vascular tone.

## 13. K_2P_13.1 (THIK-1)

K_2P_13.1 (THIK-1) mRNA was described in the CNS, arterial smooth muscle cells, the kidney and myocardial tissue samples via RT-PCR [15,22,26,66,68]. In patient-derived myocardial tissue samples, *KCNK13* mRNA abundance (TaqMan qPCR) could be demonstrated with atrial predominance (Table 2) [10]. Heterologously expressed K_2P_13.1 (THIK-1) channel homodimers were inhibited by the antiarrhythmic drugs lidocaine, mexiletine, propafenone and propranolol, while administration of quinidine, amiodarone, dronedarone or ranolazine resulted in a mild channel activation (Table 3) [82,109,129].

The observation of reduced *KCNK13* mRNA levels in patients with chronic AF or heart failure, which could also be recapitulated in a porcine large animal model of combined AF and heart failure might point towards a physiological role of K_2P_13.1 (THIK-1) currents in regulating atrial electrophysiology [10,40,129]. Finally, ventricular expression levels of *KCNK13* mRNA, were described as unchanged in heart failure patients (Table 4) [22].

## 14. K_2P_15.1 (TALK-5)

Data on cardiac expression of K_2P_15 (TASK-5) remain sparse. While some work has shown evidence of *KCNK15* mRNA abundance in rodent hearts (RT-PCR), very low levels of mRNA at best have been detected in human (northern blot, RT-PCR, TaqMan qPCR; Table 2) [10,26,48,69,70] or rodent (RT-PCR, TaqMan-qPCR) [15,16,26] heart samples by other groups. Downregulation of atrial *KCNK15* mRNA was reported in a murine CREM-TG model of AF (Table 4) [16]. Finally, functionality of K_2P_15 (TASK-5) channel subunits is still controversial, as recombinantly expressed K_2P_15 (TASK-5) homodimers do not give rise to potassium currents [8].

## 15. K_2P_16.1 (TALK-1)

K_2P_16.1 (TALK-1) is primarily expressed in pancreatic beta cells, where it is supposed to regulate insulin secretion. Recently, a gain of function mutation in *KCNK16* was identified to cause maturity-onset diabetes of the young [197]. Five studies showed low to negligible abundance of *KCNK16* mRNA in human or rat cardiac tissue samples (Table 2) [10,15,60,67,71]. Upon heterologous expression in *Xenopus laevis* oocytes, homodimeric K_2P_16.1 (TALK-1) channels are inhibited by ranolazine (Table 3) [109].

## 16. K_2P_17.1 (TALK-2)

K_2P_17.1 (TALK-2) channel subunits are expressed in the human heart (northern blot, RT-PCR, Taq-Man qPCR, western blot) [5,10,22,40,67,73,75] and in patient-derived iPSC (RT-PCR, qPCR, IF) [22,74]. Cardiac mRNA levels of *KCNK17* were described as atrial predominant with highest abundance in purkinje fibers (qPCR, Taq-Man qPCR; Table 2) [5,10]. Reports of reduced *KCNK17* mRNA levels in atrial fibrillation [10] and heart failure [22,40] suggest a role for K_2P_17.1 (TALK-2) in the pathophysiology of important cardiac pathologies. K_2P_17.1 (TALK-2) channel subunits were described to heterodimerize with atrial K_2P_3.1 (TASK-1), thereby modulating biophysical and pharmacological properties of atrial *I*_TASK-1_ [198]. In heterologous expressions systems, K_2P_17.1 (TALK-2) channel homodimers were reported to be activated by propafenone, quinidine, mexiletine, propranolol, vernakalant, and metoprolol [75]. Amiodarone, sotalol, verapamil, and ranolazine were further described to inhibit K_2P_17.1 (TALK-2) homodimers (Table 3) [75,83]. In iPSC, suppression of K_2P_17.1 (TALK-2) expression was shown to prolong APD (Table 4) [22] while overexpression of K_2P_17.1 (TALK-2) shortened APD levels in the cultured, cardiomyocyte derived HL-1 cell line [5]. Recently, a patient suffering from progressive and severe cardiac conduction disorder in combination with idiopathic ventricular fibrillation was identified to carry both, a splice site mutation in the sodium channel gene *SCN5A* as well as a mutation in the *KCNK17* gene [5]. This K_2P_17.1 (TALK-2) G88R mutation, located in the first extracellular pore loop was shown to increase K_2P_17.1 (TALK-2) currents to about three times upon heterologous expression. Overexpression of K_2P_17.1 (TALK-2) G88R in spontaneously beating HL-1 cells was shown to result in a reduction of the beating frequency, hyperpolarization of the membrane potential and a strong slowing of the upstroke velocity [5].

Single nucleotide polymorphisms in the *KCNK17* gene which increase K_2P_17.1 (TALK-2) channel subunit expression levels are associated with the occurrence of ischemic stroke in Caucasians but not in a Chinese population [137,199]. This observation links the channel once again to the pathophysiology of atrial fibrillation. *KCNK17* was further proposed as a genetic modifier of long QT syndrome type 2 severity, as a common *KCNK17* gain-of-function variant was shown to be LQTS protective by promoting APD shortening [74].

The cardiac characterization of the K_2P_17.1 (TALK-2) channel is complicated by the fact that to date no specific inhibitors are available that would allow functional studies (Table 3). Furthermore, no ortholog to the *KCNK17* gene could be identified in mice and the porcine K_2P_17.1 (TALK-2) channel subunit does not appear to show functional activity after heterologous expression in *Xenopus laevis* oocytes (unpublished observation of our lab).

## 17. K_2P_18.1 (TRESK)

*KCNK18* mRNA, encoding K_2P_18.1 (TRESK) channel subunits was detected in human spinal cord, trigeminal ganglia, and brain but not in the heart (RT-PCR and TaqMan qPCR; Table 2) [10,61,77,78]. Accordingly, K_2P_18.1 (TRESK) channels are supposed to play a key role in pain perception and *KCNK18* was identified as a potential susceptibility gene for migraine, while a cardiac role of this channel is rather unlikely [1]. TRESK channels may nevertheless exert indirect effects on the cardiovascular system: For example, high-fat diet-induced vagal afferent dysfunction has been described to be mediated via upregulation of K_2P_18.1 (TRESK) [200]. Heterologously expressed K_2P_18.1 (TRESK) channel homodimers are inhibited by lidocaine, verapamil, quinidine and apamin (Table 3) [76,200].

## 18. Conclusions

Overall, K_2P_ channels are an exciting and relevant new potassium channel class with relevance to a wide variety of disease conditions. For several members, reproducible mRNA regulation patterns in atrial fibrillation, heart failure and other cardiac disease could be described. However, the functional consequence remains difficult to assess, especially in cases where no specific channel inhibitors are available (Table 3), since surface expression and current amplitude in cardiomyocytes cannot be directly inferred from mRNA expression [11]. Further, the actual significance of the individual K_2P_ subgroups, some of which show only weak expression patterns, merits further investigation. To date, little is also known about the differential expression of K_2P_ channels in different cardiac cell populations and the consequence of remodelling in different cell types. In this regard, single cell next generation sequencing technology is expected to provide further evidence soon. Furthermore, computational models of cardiac electrophysiology must consider effects of K_2P_ channels. Taken together, emerging evidence suggests that K_2P_ channels play an important role in cardiac repolarization and in the development of various cardiac arrhythmias such as atrial fibrillation, conduction disorders, and ventricular proarrhythmia that goes far beyond the role of unspecific leak currents.

## Figures and Tables

**Table 1:** Nomenclature of K_2P_-channels.

Gene Name	IUPHAR K_2P_ Nomenclature	Functional Name	Other Names	Crystal Structure
*KCNK1*	K_2P_1.1	TWIK-1 (Tandem of P-domains in a weak inward-rectifying K^+^ channel 1)	hOHO, DPK, KCNO1	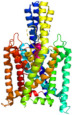
*KCNK2*	K_2P_2.1	TREK-1 (TWIK-related K^+^ channel 1)	TPKC1	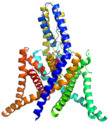
*KCNK3*	K_2P_3.1	TASK-1 (TWIK-related acid-sensitive K^+^ channel 1)	TBAK-1, OAT-1, PPH4	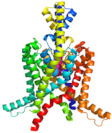
*KCNK4*	K_2P_4.1	TRAAK (TWIK-related arachidonic acid activated K^+^ channel)	FHEIG	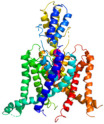
*KCNK5*	K_2P_5.1	TASK-2 (TWIK-related acid-sensitive K^+^ channel 2)		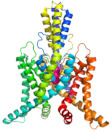
*KCNK6*	K_2P_6.1	TWIK-2 (Tandem of P-domains in a weak inward-rectifying K^+^ channel 2)	TOSS	-
*KCNK7*	K_2P_7.1	TWIK-3 (Tandem of P-domains in a weak inward-rectifying K^+^ channel 3)		-
The name *kcnk8* was initially given to a murine K_2P_ gene which was later identified as an ortholog of the human *KCNK7* gene and therefore renamed to *kcnk7*				
*KCNK9*	K_2P_9.1	TASK-3 (TWIK-related acid-sensitive K^+^ channel 3)	KT3.2, BIBARS, TASK32	-
*KCNK10*	K_2P_10.1	TREK-2 (TWIK-related K^+^ channel 2)	PPP1R97	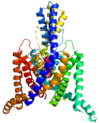
*KCNK11* was withdrawn due to nomenclature duplications with *KCNK15*				
*KCNK12*	K_2P_12.1	THIK-2 (Tandem pore domain halothane inhibited K^+^ channel 2)		-
*KCNK13*	K_2P_13.1	THIK-1 (Tandem pore domain halothane inhibited K^+^ channel 1)		-
*KCNK14* was withdrawn due to nomenclature duplications with *KCNK15*				
*KCNK15*	K_2P_15.1	TASK-5 (TWIK-related acid-sensitive K^+^ channel 5)	KT3.3, dJ781B1.1	-
*KCNK16*	K_2P_16.1	TALK-1 (TWIK-related alkaline pH-activated K^+^ channel 1)		-
*KCNK17*	K_2P_17.1	TALK-2 (TWIK-related alkaline pH-activated K^+^ channel 2)	TASK-4	-
*KCNK18*	K_2P_18.1	TRESK (TWIK-related spinal cord K^+^ channel 1)	MGR13, TRIK, TRESK2	-

IUPHAR, International Union of Basic and Clinical Pharmacology. Visualizations of the channel structurs were generated with PyMOL (TM) Molecular Graphics System, Version 2.3.0 (Schrodinger, LLC; New York, NY, USA) from crystall stuctures with the protein database enty numbers: 3UKM, 4TWK, 6RV2, 3UM7, 6WLV, 3UX0 and 4BW5.

**Table 2:** Evidence in literature for cardiac expression of K_2P_ channel subunits at mRNA or protein level in different species.

K_2P_ Channel Subunit	Species	Protein /mRNA	Observation	Citation
*K_2P_1.1* *(TWIK-1)*	Zebrafish	mRNA (RT-PCR, ISH)	Ubiquitous *kcnk1a* and *kcnk1b* ortholog mRNA in embryonic heart	[11]
	Mouse	mRNA (RT-PCR)	No cardiac mRNA abundance	[17]
	Mouse	mRNA (RT-qPCR, TaqMan)	Moderate cardiac mRNA abundance, V > A	[16]
	Rat	mRNA (RT-PCR)	Moderate cardiac mRNA abundance, A > V	[18]
	Rat	mRNA (RT-PCR)	Moderate cardiac mRNA abundance	[15]
	Rat	mRNA (RT-qPCR, TaqMan)	Cardiac mRNA abundance mRNA detected in sinoatrial tissue	[19]
	Human	mRNA (NB)	Cardiac mRNA abundance	[20]
	Human	mRNA (RT-PCR)	Cardiac mRNA abundance, V > A	[21]
	Human	mRNA (RT-qPCR, TaqMan)	Cardiac mRNA abundance, A > V Highest mRNA level among all K_2P_ channels	[10]
	Human	mRNA (RT-qPCR)	Cardiac mRNA abundance, A>V	[13]
	Human	mRNA (RT-qPCR)	mRNA detected in human ventricular tissue mRNA detected in iPS-derived cardiomyocytes	[22]
	Human	mRNA (RT-qPCR, TaqMan)	Cardiac mRNA abundance	[23]
	Human	mRNA (Affymetrix chip and RT-qPCR, TaqMan)	Cardiac mRNA abundance, A > Purkinje fibers > V	[12]
	Human	mRNA (Affymetrix chip, RT-qPCR, TaqMan) and protein (WB)	Cardiac mRNA abundance, A > Purkinje fibers > V Cardiac protein expression, A > V	[14]
*K_2P_2.1* *(TREK-2)*	Mouse	mRNA (NB)	Cardiac mRNA abundance	[24]
	Mouse	mRNA (RT-PCR)	Cardiac mRNA abundance, V > A	[17]
	Mouse	mRNA (RT-PCR)	Cardiac mRNA abundance	[25]
	Mouse	mRNA (RT-qPCR) and protein (WB)	Cardiac mRNA abundance, V > A Cardiac protein expression	[26]
	Mouse	mRNA (RT-qPCR, TaqMan)	Cardiac mRNA levels, V > A	[16]
	Mouse	Protein (IF)	Protein expression in isolated ventricular cardiomyocytes	[27]
	Rat	mRNA (RT-PCR)	mRNA abundance in isolated ventricular cardiomyocytes	[28]
	Rat	mRNA (RT-PCR)	Cardiac mRNA abundance, A and V	[18]
	Rat	mRNA (RT-PCR)	Cardiac mRNA abundance, A and V	[29]
	Rat	mRNA (RT-PCR)	Endocardial mRNA levels > epicardial mRNA expression	[30]
	Rat	mRNA (RT-PCR)	Cardiac mRNA levels, mRNA detected in cardiomyocytes	[15]
	Rat	mRNA (RT-PCR)	Cardiac mRNA abundance	[31]
	Rat	mRNA (RT-qPCR)	Cardiac mRNA abundance Cardiac mRNA adult heart > fetal heart	[18]
	Rat	mRNA (RT-qPCR, TaqMan)	Cardiac mRNA abundance in sinoatrial tissue	[19]
	Rat	mRNA (RT-PCR)and protein (IF)	Cardiac mRNA abundance, A and V Protein expression in isolated cardiomyocytes	[32]
	Rat	mRNA (RT-PCR) and protein (IF)	mRNA and protein expression in rat cardiomyocytes	[33]
	Rat	mRNA (RT-PCR) and protein (WB, IF)	Cardiac mRNA and protein expression, A and V	[34]
	Rabbit, mouse	Protein (WB)	Cardiac protein expression, SAN > A > V	[35]
	Pig	mRNA (RT-qPCR, TaqMan) and protein (WB)	Cardiac mRNA and protein expression, V = A mRNA and protein expression in sinoatrial and atrioventricular node	[36]
	Pig, human	mRNA (RT-qPCR, TaqMan)	Atrial mRNA expression in human and pig	[37]
	Human	mRNA (RT-PCR)	Cardiac mRNA abundance	[31]
	Human	mRNA (RT-qPCR)	Low cardiac mRNA abundance	[38]
	Human	mRNA (RT-qPCR)	Low cardiac mRNA abundance	[39]
	Human	mRNA (RT-qPCR)	Cardiac mRNA abundance, V Low mRNA abundance in iPS-derived cardiomyocytes	[22]
	Human	mRNA (RT-qPCR)	Cardiac mRNA abundance	[39]
	Human	mRNA (RT-qPCR, TaqMan)	Cardiac mRNA abundance, V > A	[10]
	Human	mRNA (RT-qPCR, TaqMan)	Low cardiac mRNA abundance	[23]
	Human	mRNA (RT-qPCR, TaqMan)	Cardiac mRNA levels, V > A	[40]
	Human, mouse	mRNA (RT-qPCR, TaqMan) and protein (WB)	Cardiac mRNA and protein expression in human and mice, V > A	[41]
	Human	Protein (WB)	Cardiac protein expression	[42]
	Human	Protein (WB)	Cardiac protein expression	[43]
*K_2P_3.1* *(TASK-1)*	Chicken embryo	mRNA (ISH) and protein (IF)	Cardiac mRNA and protein expression in chicken embryos	[44]
	Mouse, human	mRNA (NB)	Human and Mouse: Cardiac mRNA abundance	[45]
	Mouse	mRNA (RT-PCR)	Cardiac mRNA abundance	[17]
	Mouse	mRNA (RT-qPCR)	Cardiac mRNA levels, V > A	[26]
	Mouse	mRNA (RT-PCR) and protein (WB)	Cardiac protein expression	[25]
	Mouse, human	mRNA (RT-qPCR, TaqMan)	Cardiac mRNA expression	[46]
	Mouse	mRNA (RT-qPCR, TaqMan) and protein (WB)	Cardiac mRNA and protein expression, A and V	[16]
	Rat	mRNA (RT-qPCR, TaqMan)	Cardiac mRNA abundance in sinoatrial tissue	[19]
	Rat	mRNA (NB, RT-PCR)	Cardiac mRNA abundance, A and V	[47]
	Rat	mRNA (RT-PCR)	Cardiac mRNA abundance, cardiomyocyte mRNA abundance	[15]
	Rat	mRNA (RT-PCR)	Cardiac mRNA abundance	[48]
	Rat	mRNA (RT-PCR)	Cardiac mRNA abundance, A > V	[18]
	Rat, guinea pig, human	mRNA (RT-qPCR, TaqMan)	Human: Cardiac mRNA levels, V > A Rat: Cardiac mRNA abundance, A and V Guinea pig: Cardiac mRNA levels, V > A	[49]
	Dog	Protein (WB)	Atrial protein expression	[50]
	Pig	mRNA (RT-qPCR, TaqMan) and protein (WB)	Cardiac mRNA and protein expression mRNA abundance in sinoatrial and atrioventricular node	[51]
	Pig	mRNA (RT-qPCR, TaqMan) and protein (WB)	Cardiac mRNA and protein expression	[52]
	Human	mRNA (RT-qPCR)	Low cardiac mRNA abundance	[38]
	Human	mRNA (RT-qPCR)	mRNA abundance in Purkinje fibers	[5]
	Human	mRNA (RT-qPCR)	Cardiac mRNA abundance, A > V Cardiac mRNA adult heart > fetal heart	[39]
	Human	mRNA (RT-qPCR)	Low mRNA abundance in human ventricular tissue mRNA abundance in iPS-derived cardiomyocytes	[22]
	Human	mRNA (RT-qPCR, TaqMan)	mRNA levels in isolated atrial cardiomyocytes > in isolated atrial fibroblasts	[53]
	Human	mRNA (RT-qPCR, TaqMan)	Cardiac mRNA abundance	[23]
	Human	mRNA (RT-qPCR, TaqMan)	Cardiac mRNA levels, A > V	[54]
	Human	mRNA (Affymetrix chip and RT-qPCR, TaqMan)	Cardiac mRNA abundance, A	[55]
	Human	mRNA (Affymetrix chip and RT-qPCR, TaqMan)	Cardiac mRNA levels, A > V Expression in Purkinje fibers	[14]
	Human	mRNA (Affymetrix chip and RT-qPCR, TaqMan)	Cardiac mRNA expression, A > V	[12]
	Human	mRNA (RT-qPCR) and protein (IF)	Cardiac mRNA and protein expression	[56]
	Human	mRNA (RT-qPCR, TaqMan) and protein (WB)	Cardiac mRNA levels, A > V Cardiac protein expression, A	[40]
	Human	mRNA (RT-qPCR, TaqMan) and protein (WB)	Cardiac mRNA levels, A > V Cardiac protein expression, A	[10]
	Human	mRNA (bulk RNAseq)	Cardiac mRNA levels, A > V	[57]
*K_2P_4.1* *(TRAAK)*	Mouse	mRNA (RT-PCR, NB)	No cardiac mRNA detectable	[58]
	Mouse	mRNA (RT-qPCR)	Human: no cardiac mRNA detectable Mouse: Low cardiac mRNA abundance, A > V	[41]
	Mouse	mRNA (qRT-PCR) and protein (WB)	Cardiac mRNA abundance	[26]
	Mouse	mRNA (RT-qPCR, TaqMan)	No cardiac mRNA levels detectable	[16]
	Rat	mRNA (RT-PCR)	No cardiac mRNA levels	[15]
	Rat	mRNA (RT-PCR)	Low cardiac mRNA levels, A and V	[18]
	Human	mRNA (RT-qPCR)	mRNA abundance in human ventricular tissue mRNA abundance in iPS-derived cardiomyocytes	[22]
	Human	mRNA (RT-qPCR)	Low cardiac mRNA levels	[59]
	Human	mRNA (RT-qPCR, TaqMan)	Very low cardiac mRNA levels	[10]
	Human	mRNA (RT-qPCR, TaqMan)	No cardiac mRNA abundance	[23]
*K_2P_5.1* *(TASK-2)*	Mouse	mRNA (RT-PCR)	Cardiac mRNA abundance	[17]
	Mouse	mRNA (RT-PCR)	Cardiac mRNA levels, A and V	[26]
	Mouse	mRNA (RT-PCR)	Low cardiac mRNA abundance	[25]
	Mouse	mRNA (RT-qPCR, TaqMan)	Cardiac mRNA levels, A > V	[16]
	Rat	mRNA (NB)	No cardiac mRNA abundance	[60]
	Rat	mRNA (RT-PCR)	Low cardiac mRNA levels, A and V	[18]
	Rat	mRNA (RT-qPCR, TaqMan)	Cardiac mRNA abundance in sinoatrial tissue	[19]
	Human	mRNA (RT-PCR)	Cardiac mRNA abundance	[61]
	Human	mRNA (RT-qPCR, TaqMan)	Cardiac mRNA abundance	[23]
	Human	mRNA (RT-qPCR)	mRNA abundance in human ventricular tissue mRNA abundance in iPS-derived cardiomyocytes	[22]
	Human	mRNA (RT-qPCR)	Cardiac mRNA abundance	[56]
	Human	mRNA (RT-qPCR, TaqMan)	Cardiac mRNA levels, A > V	[10]
	Human	mRNA (Affymetrix chip and RT-qPCR, TaqMan)	Cardiac mRNA levels, A > V mRNA abundance in Purkinje fibers	[14]
	Human	mRNA (RT-qPCR) and protein (WB)	Very low cardiac mRNA levelsDetectable protein levels	[38]
*K_2P_6.1* *(TWIK-2)*	Mouse	mRNA (RT-qPCR, TaqMan)	Moderate cardiac mRNA abundance, A and V	[16]
	Mouse	mRNA (RT-qPCR) and protein (WB)	Low cardiac mRNA abundance, A and V Cardiac protein expression	[26]
	Rat	mRNA (RT-PCR)	Cardiac mRNA abundance Cardiac mRNA adult heart > fetal heart Highest mRNA abundance in right atrium	[18]
	Rat	mRNA (RT-PCR)	Cardiac mRNA abundance	[62]
	Rat	mRNA (RT-PCR)	Moderate cardiac mRNA abundance	[15]
	Rat	mRNA (RT-qPCR, TaqMan)	Cardiac mRNA abundance in sinoatrial tissue	[19]
	Human	mRNA (NB)	No cardiac mRNA abundance	[17]
	Human	mRNA (Hybridization array)	Cardiac mRNA levels, V > A	[62]
	Human	mRNA (RT-qPCR)	mRNA abundance in human ventricular tissue mRNA abundance in iPS-derived cardiomyocytes	[22]
	Human	mRNA (RT-qPCR, TaqMan)	Low cardiac mRNA abundance	[23]
	Human	mRNA (RT-qPCR, TaqMan)	Cardiac mRNA levels, V > A	[10]
*K_2P_7.1* *(TWIK-3)*	Mouse	mRNA (RT-qPCR, TaqMan)	No cardiac mRNA abundance detectable	[16]
	Human	mRNA (RT-qPCR)	Cardiac mRNA abundance	[63]
	Human	mRNA (RT-qPCR, TaqMan)	Cardiac mRNA abundance	[23]
	Human	mRNA (RT-qPCR, TaqMan)	Very low cardiac mRNA levels, A > V	[10]
*K_2P_9.1* *(TASK-3)*	Mouse	mRNA (RT-qPCR, TaqMan)	No cardiac mRNA abundance detectable	[16]
	Mouse	mRNA (RT-qPCR)	Low cardiac mRNA abundance	[26]
	Mouse	mRNA (RT-PCR)	Low cardiac mRNA abundance	[25]
	Rat	mRNA (RT-PCR)	Low cardiac mRNA abundance, A and V	[18]
	Rat	mRNA (RT-PCR)	Cardiac mRNA abundance	[48]
	Rat	mRNA (RT-PCR)	Cardiac mRNA abundance, cardiomyocyte mRNA expression	[15]
	Rat, guinea pig, human	mRNA (RT-qPCR, TaqMan)	Human: very low cardiac mRNA abundance Rat: no cardiac mRNA abundance Guinea pig: low cardiac mRNA abundance, V > A	[49]
	Guinea pig	mRNA (RT-PCR)	No cardiac mRNA abundance	[64]
	Human	mRNA (RT-qPCR)	Moderate mRNA abundance in human ventricular tissue mRNA abundance in iPS-derived cardiomyocytes	[22]
	Human	mRNA (RT-qPCR, TaqMan)	Very low cardiac mRNA levels, A > V	[10]
	Human	mRNA (RT-qPCR, TaqMan)	Cardiac mRNA abundance	[23]
	Human	mRNA (RT-qPCR) an protein (IF)	Strong cardiac mRNA and protein expression	[56]
*K_2P_10.1* *(TREK-2)*	Mouse	mRNA (RT-qPCR, TaqMan)	No cardiac mRNA abundance detectable	[16]
	Rat	mRNA (RT-PCR)	Cardiac mRNA levels, A > V	[18]
	Rat	mRNA (RT-PCR)	Moderate cardiac abundance	[15]
	Rat	mRNA (RT-PCR, NB)	No cardiac mRNA abundance	[65]
	Human	mRNA (RT-qPCR)	mRNA abundance in human ventricular tissue mRNA abundance in iPS-derived cardiomyocytes	[22]
	Human	mRNA (RT-qPCR, TaqMan)	Mild cardiac mRNA abundance, A > V	[41]
	Human	mRNA (RT-qPCR, TaqMan)	Low cardiac mRNA abundance	[23]
	Human	mRNA (RT-qPCR, TaqMan)	Low cardiac mRNA levels, A > V	[10]
*K_2P_12.1* *(THIK-2)*	Mouse	mRNA (RT-PCR)	Very low cardiac mRNA abundance	[15]
	Mouse	mRNA (RT-qPCR, TaqMan)	No cardiac mRNA levels detectable	[16]
	Rat	mRNA (RT-PCR)	No cardiac mRNA abundance	[66]
	Human	mRNA (NB)	Cardiac mRNA abundance	[67]
	Human	mRNA (RT-qPCR, TaqMan)	Very low cardiac mRNA abundance, A and V	[10]
*K_2P_13.1* *(THIK-1)*	Zebrafish	mRNA (RT-PCR)	Cardiac mRNA abundance	[68]
	Mouse	mRNA (RT-PCR)	Cardiac mRNA abundance	[15]
	Mouse	mRNA (RT-qPCR)	Cardiac mRNA abundance	[26]
	Mouse	mRNA (RT-qPCR, TaqMan)	Very low cardiac mRNA abundance	[16]
	Rat	mRNA (RT-PCR)	Cardiac mRNA abundance	[66]
	Human	mRNA (RT-qPCR)	mRNA abundance in human ventricular tissue mRNA abundance in iPS-derived cardiomyocytes	[22]
	Human	mRNA (RT-qPCR, TaqMan)	Low cardiac mRNA abundance	[23]
	Human	mRNA (RT-qPCR, TaqMan)	Low cardiac mRNA abundance, A > V	[10]
*K_2P_15.1* *(TASK-5)*	Mouse	mRNA (RT-qPCR)	Cardiac mRNA abundance	[26]
	Mouse	mRNA (RT-qPCR, TaqMan)	Very low cardiac mRNA abundance	[16]
	Rat	mRNA (RT-PCR)	No cardiac mRNA abundance	[48]
	Rat	mRNA (RT-PCR)	Moderate cardiac abundance	[15]
	Human	mRNA (RT-PCR)	Cardiac mRNA abundance	[69]
	Human	mRNA (RT-PCR, NB)	No cardiac mRNA abundance	[70]
	Human	mRNA (RT-qPCR, TaqMan)	Low cardiac mRNA abundance, A and V	[10]
*K_2P_16.1* *(TALK-1)*	Rat	mRNA (NB)	No cardiac mRNA abundance	[60]
	Rat	mRNA (RT-PCR)	Moderate cardiac abundance	[15]
	Human	mRNA (NB)	No cardiac mRNA abundance	[67]
	Human	mRNA (RT-PCR, NB)	No cardiac mRNA abundance	[71]
	Human	mRNA (RT-qPCR, TaqMan)	Very low cardiac mRNA abundance, A and V	[10]
*K_2P_17.1* *(TALK-2)*	Zebrafish	mRNA (RT-PCR)	No cardiac abundance	[72]
	Rat	mRNA (NB)	Cardiac mRNA abundance	[60]
	Human	mRNA (NB)	Cardiac mRNA abundance	[67]
	Human	mRNA (RT-PCR)	Cardiac mRNA levels, A > V	[73]
	Human	mRNA (RT-qPCR)	mRNA abundance in human ventricular tissue mRNA abundance in iPS-derived cardiomyocytes	[22]
	Human	mRNA (RT-qPCR)	Cardiac mRNA abundance	[56]
	Human	mRNA (RT-qPCR)	Cardiac mRNA abundance mRNA abundance in sinoatrial and atrioventricular node Purkinje fibers > A > V	[5]
	Human	mRNA (single cell RT-qPCR) and protein (IF)	mRNA and protein abundance in iPS-derived cardiomyocytes	[74]
	Human	mRNA (RT-qPCR, TaqMan)	Cardiac mRNA levels, A > V	[10]
	Human	mRNA (RT-qPCR, TaqMan) and protein (WB)	Cardiac mRNA and protein expression	[40]
	Human	Protein (WB)	Cardiac protein expression, A	[75]
*K_2P_18.1* *(TRESK)*	Zebrafish	mRNA (ISH)	No cardiac abundance	[76]
	Mouse	mRNA (RT-qPCR, TaqMan)	Very low cardiac mRNA abundance	[77]
	Mouse	mRNA (RT-qPCR, TaqMan)	Very low cardiac mRNA abundance	[16]
	Human	mRNA (RT-PCR)	No cardiac mRNA abundance	[61]
	Human	mRNA (RT-PCR)	No cardiac mRNA abundance	[78]
	Human	mRNA (RT-qPCR, TaqMan)	Very low cardiac mRNA abundance	[10]

A, expression in atrial tissue; IF, immunofluorescence; iPS, induced pluripotent stem cell; ISH, in situ hybridization; LA, left atrium; NB, Northern blot; RT-PCR, reverse transcriptase PCR; RT-qPCR, reverse transcriptase quantitative PCR; RA, right atrium; TAC, transverse aortic constriction; TaqMan, reverse transcriptase quantitative PCR employing TaqMan^®^ hydrolyse probes to increase specificity; V, expression in ventricular tissue; WB, Western blot.

**Table 3:** Pharmacological profile of K_2P_-channels.

K_2P_ Channel	Drug/Compound	Effect (Organism)	EC_50_ /IC_50_ (Organism)	Citation
*K_2P_1.1* *(TWIK-1)*	Quinine	Inhibition (XO)	50 µM (XO)	[20]
	Quinidine	Inhibition (XO)	95 µM (XO)	[20]
	Barium	Inhibition (XO)	100 µM (XO)	[20]
	Charybdotoxin	< 10% inhibition at 3 nM (XO)	n.m.	[20]
	Dendrotoxin	< 10% inhibition at 100 nM (XO)	n.m.	[20]
	Apamin	< 10% inhibition at 300 nM (XO)	n.m.	[20]
	Clofilium	< 10% inhibition at 30 µM (XO)	n.m.	[20]
	Glibenclamid	< 10% inhibition at 30 µM (XO)	n.m.	[20]
	Cromakalim	No effect at 100 µM (XO)	n.m.	[20]
	Tedisamil	30% inhibition at 100 µM (XO)	n.m.	[20]
	Dronedarone	No significant effect at 100 µM (XO)	n.m.	[82]
	Amiodarone	< 10% inhibition at 100 µM (XO)	n.m.	[20]
	Pinacidil	No effect at 100 µM (XO)	n.m.	[20]
	Vernakalant	No significant effect at 100 µM (XO)	n.m.	[83]
	Flecainide	No significant effect at 100 µM (XO)	n.m.	[84]
	Genistein	No significant effect at 100 µM (XO)	n.m.	[85]
	4-AP	< 10% inhibition at 1 mM (XO)	n.m.	[20]
	TEA	30% inhibition at 10 mM (XO)	n.m.	[20]
*K_2P_2.1* *(TREK-1)*	**GI-530159**	**High affinity K_2P_2.1 activator (MC)**	**890 nM (MC)**	[86]
	Copper	Activation (MC)	3 µM (MC)	[87]
	Ostruthin	Activator (MC)	5.3 µM (MC)	[88]
	**BL-1249**	**High affinity TREK-1/2 activator (XO)**	**5.5 µM (XO)**	[89]
	**ML402**	**High affinity TREK-1/2 activator (XO)**	**13.7 µM (XO)**	[90]
	**ML335**	**High affinity TREK-1/2 activator (XO)**	**14.3 µM (XO)**	[90]
	**ML67-33**	**High affinity TREK-1/2 activator (XO)**	**36.3 µM (XO); 9.7 µM (MC)**	[91]
	Pranlukast	66.4% activation at 3 µM (MC)	n.m.	[92]
	DCPIB	~3-fold activation at 10 µM (MC)	n.m.	[93]
	Morphine	~2-fold activation at 10 µM (MC)	n.m.	[94]
	Flufenamic acid	~4-fold activation at 100 µM (MC)	n.m.	[95]
	Niflumic acid	~2.5-fold activation at 100 µM (MC)	n.m.	[95]
	Mefenamic acid	~2-fold activation at 100 µM (MC)	n.m.	[95]
	Carbamazepine	42% activation at 100 µM (MC)	n.m.	[96]
	Valproate	28% activation at 100 µM (MC)	n.m.	[96]
	Gabapentin	25% activation at 100 µM (MC)	n.m.	[96]
	Diethyl ether	~1.75-fold activation at 600 µM (MC)	n.m.	[97]
	Chloroform	~3.5-fold activation at 800 µM (MC)	n.m.	[97]
	Lithium	31% activation at 1 mM (MC)	n.m.	[96]
	Rubidium	27% activation at 1 mM (MC)	n.m.	[96]
	Halothane	~1.4-fold activation at 1 mM (MC)	n.m.	[97]
	Isoflurane	~1.5-fold activation at 2 mM (MC)	n.m.	[97]
	Cyclopropane	~30% activation at 10% (MC)	n.m.	[98]
	Xenon	~30% activation at 80% (MC)	n.m.	[98]
	Nitrous oxide	~30% activation at 80% (MC)	n.m.	[98]
	**Spadin**	**High affinity K_2P_2.1 inhibitor (MC)**	**40 nM (MC)**	[99]
	Amlodipin	Inhibition (MC)	430 nM (MC)	[100]
	Nigludipine	Inhibition (MC)	750 nM (MC)	[100]
	Pimozide	Inhibition (MC)	1.8 µM (MC)	[101]
	Fluphenthixol	Inhibition (MC)	2.0 µM (MC)	[101]
	Chlorpromazine	Inhibition (MC)	2.7 µM (MC)	[96,101]
	Sipatrigine	73.3% inhibition at 10 µM (MC)	4 µM	[59]
	Fluphenazine	Inhibition (MC)	4.7 µM (MC)	[101]
	Haloperidol	Inhibition (MC)	5.5 µM (MC)	[101]
	Norfluoxetine	Inhibition (MC)	9 µM (MC)	[102]
	Vernakalant	Inhibition (MC)	13.3 µM (MC)	[84]
	Loxapine	Inhibition (MC)	19.7 µM (MC)	[101]
	Fluoxetine	Inhibition (MC)	19–37.9 µM (MC)	[96,102]
	Carvedilol	Inhibition (XO, MC)	20.3 μM (XO); 1.6 μM (MC)	[42]
	A1899 *(High affinity K_2P_3.1 inhibitor)*	Inhibition (XO)	23.8 µM (XO)	[103]
	Dronedarone	Inhibition (XO, MC)	26.7 μM (XO); 6.1 μM (MC)	[82]
	Propafenone	Inhibition (XO, MC)	51.0 μM (XO); 7.9 μM (MC)	[104]
	Levobupivacaine	Inhibition (MC)	126 µM (MC)	[105]
	Diltiazem	Inhibitor (MC)	180 µM (MC)	[95]
	Lidocaine	Inhibition (MC)	207 μM (MC)	[106]
	Bupivacaine	Inhibition (MC)	370 µM (MC)	[107]
	Caffeine	Inhibition (MC)	377 µM (MC)	[108]
	Ropivacaine	Inhibition (MC)	402 µM (MC)	[105]
	Theophylline	Inhibition (MC)	486 µM (MC)	[108]
	Zinc	Inhibition (MC)	659 µM (MC)	[87]
	Mexiletine	Inhibition (XO, MC)	1.3 mM (XO); 182 μM (MC);	[104]
	Tetramethyl-ammonium	63% inhibition (MC)	n.m.	[24]
	Lamotrigine	~10% inhibition at 10 µM (MC)	n.m.	[59]
	Metoprolol	~20% inhibition at 100 µM (XO)	n.m.	[42]
	Propranolol	~30% inhibition at 100 µM (XO)	n.m.	[42]
	Citalopram	59% inhibition at 100 µM (MC)	n.m.	[96]
	Barium	50% inhibition at 300 µM (XO)	n.m.	[24]
	Ranolazine	7.35% inhibition at 300 µM (XO)	n.m.	[109]
	Clozapine	Inhibition (MC)	n.m.	[101]
	Sulpiride	No significant effect at 10 µM (MC)	n.m.	[101]
	Tiapride	No significant effect at 10 µM (MC)	n.m.	[101]
	Glibenclamide	No significant effect at 10 µM (XO)	n.m.	[24]
	Cesium	No significant effect at 100 µM (XO)	n.m.	[24]
	Gadolineum	No significant effect at 100 µM (XO)	n.m.	[24]
	TEA	No significant effect at 100 µM (XO)	n.m.	[24]
	Quinine	No significant effect at 100 µM (XO)	n.m.	[24]
	Quinidine	No significant effect at 100 µM (XO)	n.m.	[24]
	Tedisamil	No significant effect at 100 µM (XO)	n.m.	[24]
	Genistein	No significant effect at 100 µM (XO)	n.m.	[85]
	Flecainide	No significant effect at 100 µM (XO, MC)	n.m.	[84]
	Amiodarone	No significant effect (XO)	n.m.	[110]
	Sotalol	No significant effect (XO)	n.m.	[82]
	Digoxin	No significant effect (XO)	n.m.	[111]
	Digitoxin	No significant effect (XO)	n.m.	[111]
	A293	No significant effect (XO)	n.m.	[10]
	Ajmaline	No significant effect (MC)	n.m.	[104]
	GsMTx4	No significant effect (MC)	n.m.	[112]
	Magnesium	No significant effect (XO)	n.m.	[24]
*K_2P_3.1* *(TASK-1)*	Halothane	Activation (XO, MC)	300–1000 µM (XO)	[97,113,114]
	Sevoflurane	~40% activation at 1 mM	n.m.	[114]
	Isoflurane	~15% activation at 1 mM (XO) ~20% activation at 2 mM (MC)	n.m.	[97,113]
	**BAY2341237**	**High affinity K_2P_3.1 inhibitor**	**7.6 nM (XO)**	[115]
	**BAY1000493**	**High affinity K_2P_3.1 inhibitor**	**9.5 nM (XO)**	[115]
	**ML365**	**High affinity K_2P_3.1 inhibitor**	**16 nM (MC)**	[116]
	**A1899 (S20951)**	**High affinity K_2P_3.1 inhibitor**	**35 nM (XO); 7 nM (MC)**	[103,115]
	S9947 *(K_V_1.5 blocker)*	Inhibition (XO)	200 nM (XO)	[103,117]
	**A293** **(AVE1231)**	**High affinity K_2P_3.1 inhibitor**	**222 nM (XO)**	[10,15]
	PK-THPP	Inhibition (XO)	243 nM	[118]
	MSD-D *(K_V_1.5 blocker)*	Inhibition (XO)	350 nM (XO)	[117]
	Amiodarone	Inhibition (XO)	400 nM (XO)	[82,110]
	Doxapram	Inhibition (XO, MC)	410 nM (XO)	[119]
	AVE0118 *(K_V_1.5 blocker)*	Inhibition (XO)	600 nM (XO)	[117]
	Methanandamide	Inhibition (XO)	700 nM (MC)	[120]
	Digoxin	Inhibition (XO)	900 nM (XO)	[111]
	ICAGEN-4 *(K_V_1.5 blocker)*	Inhibition (XO)	1.05 µM (XO)	[117]
	ML308 *(High affinity K_2P_9.1 inhibitor)*	Inhibition (MC)	3.2 µM (MC)	[121]
	Carvedilol	Inhibition (XO, MC)	3.8 µM (XO); 0.83 µM (MC)	[42]
	Digitoxin	Inhibition (XO)	7.4 µM (XO)	[111]
	Genistein	81.1% inhibition at 100 µM (XO)	12.3 µM (MC)	[85]
	Dronedarone	Inhibition (XO, MC)	18.7 µM (XO); 5.2 µM (MC)	[82]
	Propafenone	Inhibition (XO, MC)	18.1 μM (XO); 5.1 μM (MC);	[104]
	Etidocaine	Inhibition (XO)	39 µM (XO)	[122]
	Ostruthin	Inhibition (MC)	41 µM (MC)	[88]
	R-Ropivacaine	Inhibition (XO)	51 µM (XO)	[122]
	S-Ropivacaine	Inhibition (XO)	53 µM (XO)	[122]
	Bupivacaine	Inhibition (XO)	68 µM (XO)	[123]
	Etomidate	Inhibition (XO)	119 µM (XO)	[113]
	Zinc	Inhibition (XO)	175 µM (XO)	[123]
	Ranolazine	Inhibition (XO, MC)	198.4 µM (XO); 30.6 µM (MC)	[109]
	Lidocain	Inhibition (XO)	222 µM (XO)	[122]
	Mexiletine	Inhibition (XO, MC)	405 µM (XO); 97.3 μM (MC)	[104]
	Tetracaine	Inhibition (XO)	668 µM	[122]
	Mepivacaine	Inhibition (XO)	709 µM (XO)	[122]
	Agitoxin	< 15%inhibition at 1 nM (XO)	n.m.	[123]
	Margatoxin	< 15%inhibition at 10 nM (XO)	n.m.	[123]
	Dendrotoxin	< 15%inhibition at 100 nM (XO)	n.m.	[123]
	Charybdotoxin	< 15%inhibition at 200 nM (XO)	n.m.	[123]
	Anandamide	~90% inhibition at 3 µM (MC)	n.m.	[120]
	CP55940 (CB1/CB2agonist)	~50% inhibition at 10 µM (MC)	n.m.	[120]
	Sipatrigine	37%inhibition at 10 µM (MC)	n.m.	[59]
	Glibenclamid	< 15%inhibition at 30 µM (XO)	n.m.	[123]
	Propranolol	~60% inhibition at 100 µM (XO)	n.m.	[42]
	Cesium	31% inhibition at 100 µM (XO)	n.m.	[45]
	Quinidine	< 20–71 % inhibition at 100 µM (XO)	n.m.	[45,123]
	Quinine	< 20 % inhibition at 100 µM (XO)	n.m.	[45]
	Quinacrine	< 20% inhibition at 100 µM (XO)	n.m.	[45]
	Barium	~19% inhibition at 100 µM (XO)	n.m.	[45]
	Daidzein	18.2% inhibition at 100 µM (XO)	n.m.	[85]
	Cromakalim	< 15%inhibition at 100 µM (XO)	n.m.	[123]
	Metoprolol	~10% inhibition at 100 µM (XO)	n.m.	[42]
	Phenytoin	~50% inhibition at 200 µM (XO)	n.m.	[123]
	Diethyl ether	~45 % at 600 µM (MC)	n.m.	[97]
	Magnesium	~14% inhibition at 10 mM (XO)	n.m.	[123]
	4-AP	<15%inhibition at 10 mM (XO)	n.m.	[45,123]
	Flecainide	No significant effect at 100 µM (XO, MC)	n.m.	[84]
	Ouabain	No significant effect at 100 µM (XO)	n.m.	[111]
	Vernakalant	No significant effect at 100 µM (XO, MC)	n.m.	[84]
	Sotalol	No significant effect at 100 µM (XO)	n.m.	[82]
	Genistin	No significant effect at 100 µM (XO)	n.m.	[85]
	Propofol	No significant effect at 200 µM (XO)	n.m.	[113]
	Chloroform	No significant effect at 800 µM (MC)	n.m.	[97]
	TEA	No significant effect at 1 mM (XO)	n.m.	[45]
*K_2P_4.1* *(TRAAK)*	Sipatrigine	45%inhibition at 10 µM (MC)	10 µM	[59]
	ML67-33 *(High affinity TREK-1/2 activator)*	Activation (XO, MC)	27.3 µM (XO); 1.8 µM (MC)	[91]
	BL-1249 *(High affinity TREK-1/2 activator)*	Activation (XO)	48 µM (XO)	[89]
	A1899 *(High affinity K_2P_3.1 inhibitor)*	Inhibition (XO)	>20 µM (XO)	[103]
	Docosahexaenoate	~12-fold activation at 10 µM (MC)	n.m.	[58]
	Eicosapentaenoate	~8-fold activation at 10 µM (MC)	n.m.	[58]
	Arachidonic acid	~5-fold activation at 10 µM (MC)	n.m.	[58]
	Oleate	~1.5-fold activation at 10 µM (MC)	n.m.	[58]
	Linoleate	~1.5-fold activation at 10 µM (MC)	n.m.	[58]
	Riluzole	3.9-fold activation at 100 µM (MC)	n.m.	[58]
	Flufenamic acid	~2-fold activation at 100 µM (MC)	n.m.	[95]
	Niflumic acid	~2-fold activation at 100 µM (MC)	n.m.	[95]
	Mefenamic acid	~1.6-fold activation at 100 µM (MC)	n.m.	[95]
	Lamotrigine	~10% inhibition at 10 µM (MC)	n.m.	[59]
	Vernakalant	17.1% inhibition at 100 µM (XO)	n.m.	[83]
	Barium	56.7% inhibition at 1 mM (XO)	n.m.	[58]
	Charybdotoxin	No significant effect at 20 nM (XO)	n.m.	[58]
	Dendrotoxin	No significant effect at 100 nM (XO)	n.m.	[58]
	Tetrodotoxin	No significant effect at 1 µM (XO)	n.m.	[58]
	Tedisamil	No significant effect at 10 µM (XO)	n.m.	[58]
	Palmitate	No significant effect at 10 µM (MC)	n.m.	[58]
	Stearate	No significant effect at 10 µM (MC)	n.m.	[58]
	Arachidate	No significant effect at 10 µM (MC)	n.m.	[58]
	Fluphenazine	No significant effect at 10 µM (MC)	n.m.	[101]
	Chlorpromazine	No significant effect at 10 µM (MC)	n.m.	[101]
	Haloperidol	No significant effect at 10 µM (MC)	n.m.	[101]
	Fluphenthixol	No significant effect at 10 µM (MC)	n.m.	[101]
	Loxapine	No significant effect at 10 µM (MC)	n.m.	[101]
	Pimozide	No significant effect at 10 µM (MC)	n.m.	[101]
	Clozapine	No significant effect at 10 µM (MC)	n.m.	[101]
	Sulpiride	No significant effect at 10 µM (MC)	n.m.	[101]
	Tiapride	No significant effect at 10 µM (MC)	n.m.	[101]
	Tolbutamide	No significant effect at 100 µM (XO)	n.m.	[58]
	Pinacidil	No significant effect at 100 µM (XO)	n.m.	[58]
	P1060	No significant effect at 100 µM (XO)	n.m.	[58]
	Glibenclamide	No significant effect at 200 µM (XO)	n.m.	[58]
	Cobalt	No significant effect at 500 µM (XO)	n.m.	[58]
	Dronedarone	No significant effect at 100 µM (XO)	n.m.	[82]
	Flecainide	No significant effect at 100 µM (XO)	n.m.	[84]
	Genistein	No significant effect at 100 µM (XO)	n.m.	[85]
	Ranolazine	3.32 % inhibition at 300 µM (XO)	n.m.	[109]
	Diethyl ether	No significant effect at 600 µM (MC)	n.m.	[97]
	Chloroform	No significant effect at 800 µM (MC)	n.m.	[97]
	Halothane	No significant effect at 1 mM (MC)	n.m.	[97]
	Diltiazem	No significant effect at 1 mM (MC)	n.m.	[95]
	TEA	No significant effect at 1 mM (XO)	n.m.	[58]
	4-AP	No significant effect at 1 mM (XO)	n.m.	[58]
	Caesium	No significant effect at 1 mM (XO)	n.m.	[58]
	Isoflurane	No significant effect at 2 mM (MC)	n.m.	[97]
	Digoxin	No significant effect (XO)	n.m.	[111]
	Digitoxin	No significant effect (XO)	n.m.	[111]
*K_2P_5.1* *(TASK-2)*	A293 *(High affinity K_2P_3.1 inhibitor)*	Inhibition (XO)	8.1 nM (XO)	[10,15]
	A1899 *(High affinity K_2P_3.1 inhibitor)*	Inhibition (XO)	12 µM (XO)	[103]
	Quinine	Inhibition (XO)	22.4 µM (XO)	[17]
	Quinidine	65% inhibition at 100 µM (XO)	n.m.	[17]
	Zinc	15.3% inhibition at 100 µM (XO)	n.m.	[17]
	Ranolazine	30.02% inhibition at 300 µM (XO)	n.m.	[17]
	Barium	16.9% inhibition at 1 mM (XO)	n.m.	[17]
	Lidocaine	60.4% inhibition at 10 mM (XO)	n.m.	[17]
	Bupivacaine	80.9% inhibition at 10 mM (XO)	n.m.	[17]
	Arachidonic acid	No significant effect at 10 µM (XO)	n.m.	[17]
	4-AP	No significant effect at 100 µM (XO)	n.m.	[17]
	Dronedarone	No significant effect at 100 µM (XO)	n.m.	[82]
	Flecainide	No significant effect at 100 µM (XO)	n.m.	[84]
	Genistein	No significant effect at 100 µM (XO)	n.m.	[85]
	Vernakalant	No significant effect at 100 µM (XO)	n.m.	[83]
	Digoxin	No significant effect (XO)	n.m.	[111]
	Digitoxin	No significant effect (XO)	n.m.	[111]
	TEA	No significant effect at 1 mM (XO)	n.m.	[17]
	Cesium	No effect at 1 mM (XO)	n.m.	[17]
*K_2P_6.1* *(TWIK-2)*	Barium	Inhibition (MC)	~100 µM (MC)	[124]
	Quinidine	73% inhibition at 100 µM (XO)	n.m.	[124]
	Quinine	73% inhibition at 100 µM (XO)	n.m.	[124]
	Genistein	~30% inhibition at 100 µM (XO)	n.m.	[85]
	Dronedarone	10.7% inhibition at 100 µM (XO)	n.m.	[82]
	Chloroform	32% inhibition at 300 µM (XO)	n.m.	[124]
	Halothane	27% inhibition at 750 µM (XO)	n.m.	[124]
	Cesium	92% inhibition of inward current at 10 mM (XO)	n.m.	[124]
	TEA	No significant effect at 5 mM (XO)	n.m.	[124]
	4-AP	No significant effect at 3 mM (XO)	n.m.	[124]
	Glibenclamide	No significant effect at 10 µM (XO)	n.m.	[124]
	Vernakalant	No significant effect at 100 µM (XO)	n.m.	[83]
	Flecainide	No significant effect at 100 µM (XO)	n.m.	[84]
*K_2P_7.1* *(TWIK-3)*		*Non-functional channel*		
*K_2P_9.1* *(TASK-3)*	DCPIB	~3-fold activation at 10 µM (MC)	n.m.	[93]
	Halothane	65.6% activation at 1 mM (XO)	n.m.	[125]
	BAY2341237 *(High affinity K_2P_3.1 inhibitor)*	Inhibition (XO)	2.3 nM (XO)	[115]
	BAY1000493 *(High affinity K_2P_3.1 inhibitor)*	Inhibition (XO)	15.1 nM (XO)	[115]
	A1899 *(High affinity K_2P_3.1 inhibitor)*	Inhibition (XO, MC)	318 nM (XO); 70 nM (MC)	[103]
	**ML308**	**High affinity K_2P_9.1 inhibitor**	**413 nM (MC)**	[121]
	A293 *(High affinity K_2P_3.1 inhibitor)*	Inhibition (XO)	950 nM (XO)	[10,15]
	ML365 *(High affinity K_2P_3.1 inhibitor)*	Inhibition (MC)	990 nM (MC)	[116]
	Copper	Inhibition (MC)	2.7 µM (MC)	[87]
	Zinc	Inhibition (MC)	12.7 µM (MC)	[87]
	Mibefradil	Inhibition (MC)	24.6 μM (MC)	[126]
	Doxapram	Inhibition (XO)	37 µM (XO)	[119]
	L-703,606 oxalate	Inhibition (MC)	45.5 μM (MC)	[126]
	Oligomycine A	Inhibition (MC)	47.7 μM (MC)	[126]
	GW2974	Inhibition (MC)	50.1 µM (MC)	[126]
	Loratadine	Inhibition (MC)	63.4 µM (MC)	[126]
	Dihydro-β-erythroidine hydrobromide	Inhibition (MC)	73.8 µM (MC)	[126]
	(±)-Octoclothepin maleate	Inhibition (MC)	73.8 µM (MC)	[126]
	Ruthenium red	Inhibitor (XO)	114 µM	[127]
	Etomidate	Inhibition (XO)	128 µM (XO)	[113]
	Mevastatin	Inhibition (MC)	159 μM (MC)	[126]
	Ostruthin	Inhibition (MC)	227 µM (MC)	[88]
	Barium	11% inhibition at 100 µM (XO)	290 µM (XO)	[64]
	Arachidonic acid	4.81% inhibition at 10 µM (XO)	n.m.	[125]
	Genistein	~60% inhibition at 100 µM (XO)	n.m.	[85]
	Bupivacaine	50.2–56% inhibition at 100 µM (XO, MC)	n.m.	[70,125]
	Alphaxolone	49.2% inhibition at 100 µM (XO)	n.m.	[125]
	Quinidine	42.2% inhibition at 100 µM (XO)	n.m.	[125]
	Quinine	36.9% inhibition at 100 µM (XO)	n.m.	[125]
	Dronedarone	31.7% inhibition at 100 µM (XO)	n.m.	[82]
	Fluoxetine	31%inhibition at 100 µM (MC)	n.m.	[102]
	Ketamine	7.3% inhibition at 100 µM (XO)	n.m.	[125]
	Pentobarbital	4.3% inhibition at 100 µM (XO)	n.m.	[125]
	Glibenclamide	3.6% inhibition at 100 µM (XO)	n.m.	[125]
	Ranolazine	28.28% inhibition at 300 µM (XO)	n.m.	[109]
	TEA	6% inhibition at 1 mM (XO)	n.m.	[125]
	Xenon	No significant effect at 80% (MC)	n.m.	[98]
	Nitrous oxide	No significant effect at 80% (MC)	n.m.	[98]
	Cyclopropane	No significant effect at 10% (MC)	n.m.	[98]
	Propofol	No significant effect at 200 µM (XO)	n.m.	[113]
	Vernakalant	No significant effect at 100 µM (XO)	n.m.	[83]
	Flecainide	No significant effect at 100 µM (XO)	n.m.	[84]
	Digoxin	No significant effect (XO)	n.m.	[111]
	Digitoxin	No significant effect (XO)	n.m.	[111]
	Cesium	8–12% inhibition at 10 mM (XO)	n.m.	[64,125]
*K_2P_10.1* *(TREK-2)*	Ostruthin	Activator (MC)	3.7 µM (MC)	[88]
	**ML335**	**High affinity TREK-1/2 activator**	**5.2 µM (XO)**	[90]
	**ML402**	**High affinity TREK-1/2 activator**	**5.9 µM (XO)**	[90]
	Arachidonic acid	Activation (MC)	7.3 µM (MC)	[65]
	**BL-1249**	**High affinity TREK-1/2 activator**	**8.0 µM (XO)**	[89]
	**ML67-33**	**High affinity TREK-1/2 activator**	**30.2 µM (XO); 1.6 µM (MC)**	[91]
	11-deoxyprostaglandin F2α	~5-fold activation at 2 µM (MC)	n.m.	[128]
	Pranlukast	228 % activation at 3 µM (MC)	n.m.	[92]
	Ocosahexaenoicacid	~5-fold activation at 20 µM (MC)	n.m.	[65]
	Linolenic acid	~6-fold activation at 20 µM (MC)	n.m.	[65]
	Eicosapentaenoic acid	~8-fold activation at 20 µM (MC)	n.m.	[65]
	Linoleic acid	~8-fold activation at 20 µM (MC)	n.m.	[65]
	Flufenamic acid	~4-fold activation at 100 µM (MC)	n.m.	[95]
	Niflumic acid	~2.5-fold activation at 100 µM (MC)	n.m.	[95]
	Mefenamic acid	~2-fold activation at 100 µM (MC)	n.m.	[95]
	Ruthenium red	Inhibition (XO)	230 nM (XO)	[127]
	A1899 *(High affinity K_2P_3.1 inhibitor)*	Inhibition (XO)	8.4 µM (XO)	[103]
	Carvedilol	Inhibition (XO, MC)	24 µM (XO); 7.6 (MC)	[43]
	Fluoxetine	68% inhibition at 10 µM (MC)	28.7 µM (MC)	[96]
	Diltiazem	Inhibition (MC)	330 µM (MC)	[95]
	Fluphenthixol	~80% inhibition at 10 µM (MC)	n.m.	[101]
	Pimozide	~80% inhibition at 10 µM (MC)	n.m.	[101]
	Fluphenazine	~70% inhibition at 10 µM (MC)	n.m.	[101]
	Clozapine	~50% inhibition at 10 µM (MC)	n.m.	[101]
	Loxapine	~50% inhibition at 10 µM (MC)	n.m.	[101]
	Haloperidol	~50% inhibition at 10 µM (MC)	n.m.	[101]
	Paroxetin	33% inhibition at 20 µM (MC)	n.m.	[96]
	Citalopram	59% inhibition at 100 µM (MC)	n.m.	[96]
	Chlorpromazine	57% inhibition at 100 µM (MC)	n.m.	[96,101]
	Vernakalant	19.8% inhibition at 100 µM (XO)	n.m.	[83]
	Barium	36% inhibition at 2 mM (MC)	n.m.	[65]
	Sulpiride	No significant effect at 10 µM (MC)	n.m.	[101]
	Tiapride	No significant effect at 10 µM (MC)	n.m.	[101]
	Elaidic acid	No significant effect at 20 µM (MC)	n.m.	[65]
	Stearic acid	No significant effect at 100 µM (MC)	n.m.	[65]
	Palmitic acid	No significant effect at 100 µM (MC)	n.m.	[65]
	Gabapentin	No significant effect at 100 µM (MC)	n.m.	[96]
	Valproate	No significant effect at 100 µM (MC)	n.m.	[96]
	Carbamazepine	No significant effect at 100 µM (MC)	n.m.	[96]
	Flecainide	No significant effect at 100 µM (XO)	n.m.	[84]
	Genistein	No significant effect at 100 µM (XO)	n.m.	[85]
	Dronedarone	No significant effect at 100 µM (XO)	n.m.	[82]
	Quinidine	No significant effect at 100 µM (MC)	n.m.	[65]
	Bupivacaine	No significant effect at 100 µM (MC)	n.m.	[65]
	Gadolinium	No significant effect at 100 µM (MC)	n.m.	[65]
	Ranolazine	No significant effect at 300 µM (XO)	n.m.	[109]
	TEA	No significant effect at 1 mM (MC)	n.m.	[65]
	Lidocaine	No significant effect at 1 mM (MC)	n.m.	[65]
	Lithium	No significant effect at 1 mM (MC)	n.m.	[96]
	Rubidium	No significant effect at 1 mM (MC)	n.m.	[96]
	Digitoxin	No significant effect (XO)	n.m.	[111]
	Digoxin	No significant effect (XO)	n.m.	[111]
*K_2P_12.1*	Quinidine	Inhibition (XO)	160 µM (XO)	[8]
	Halothane	~50% inhibition at 5 mM (XO)	n.m.	[8]
	Arachidonic acid	No significant effect at 5 µM (XO)	n.m.	[8]
*K_2P_13.1* *(THIK-1)*	Lysophos-phatidylcholine	~20% activation at 10 µM (XO)	n.m.	[66]
	Arachidonic acid	69.6–85% activation at 5–20 µM (XO)	980 nM (XO)	[66,68]
	Dronedarone	14.9% activation at 100 µM (XO)	n.m.	[82]
	Quinidine	10.9% activation at 100 µM (XO)	n.m.	[129]
	Amiodarone	9.3% activation at 100 µM	n.m.	[129]
	Ranolazine	4.98% activation at 300 µM (XO)	n.m.	[109]
	A1899 *(High affinity K_2P_3.1 inhibitor)*	Inhibition (XO)	2.2 µM (XO)	[103]
	Mexiletine	74.6% inhibition at 1.5 mM (XO)	356 µM (XO)	[68,129]
	Halothane	56% inhibition at 5 mM (XO)	2.8 mM (XO)	[66]
	Lidocaine	59.2% inhibition at 100 µM (XO)	n.m.	[68]
	Carvedilol	No significant effect at 100 µM (XO)	n.m.	[129]
	Metoprolol	No significant effect at 100 µM (XO)	n.m.	[129]
	Vernakalant	No significant effect at 100 µM (XO)	n.m.	[83]
	Flecainide	No significant effect at 100 µM (XO)	n.m.	[84]
	Verapamil	No significant effect at 100 µM (XO)	n.m.	[129]
	Propafenone	26% inhibition at 100 µM (XO)	n.m.	[129]
	Genistein	~20% inhibition at 100 µM (XO)	n.m.	[85]
	Propranolol	37.6% inhibition at 200 µM (XO)	n.m.	[129]
	Chloroform	No significant effect at 1 mM (XO)	n.m.	[66]
	Barium	88.7% inhibition at 2 mM (XO)	n.m.	[66,68]
	Digoxin	No significant effect (XO)	n.m.	[111]
	Digitoxin	No significant effect (XO)	n.m.	[111]
*K_2P_15.1* *(TASK-5)*		*Non-functional channel*		
*K_2P_16.1* *(THIK-1)*	Digitoxin	~30% inhibition at 100 µM (XO)	n.m.	[111]
	Ranolazine	23.04% inhibition at 300 µM (XO)	n.m.	[109]
	Halothane	26.8% inhibition at 800 µM (XO)	n.m.	[67]
	Chloroform	21.5% inhibition at 800 µM (XO)	n.m.	[67]
	Barium	51.4% inhibition at 1 mM (XO)	n.m.	[67]
	Quinine	45.1% inhibition at 1 mM (XO)	n.m.	[67]
	Quinidine	36.8% inhibition at 1 mM (XO)	n.m.	[67]
	TEA	14.9% inhibition at 1 mM (XO)	n.m.	[67]
	Arachidonic acid	No significant effect at 20 µM (XO)	n.m.	[67]
	4-AP	No significant effect at 100 µM (XO)	n.m.	[67]
	Vernakalant	No significant effect at 100 µM (XO)	n.m.	[83]
	Flecainide	No significant effect at 100 µM (XO)	n.m.	[84]
	Genistein	No significant effect at 100 µM (XO)	n.m.	[85]
	Dronedarone	No significant effect at 100 µM (XO)	n.m.	[82]
	Isoflurane	No significant effect at 800 µM (XO)	n.m.	[67]
	Cesium	No significant effect at 1 mM (XO)	n.m.	[67]
	Digoxin	No significant effect (XO)	n.m.	[111]
*K_2P_17.1* *(THIK-2)*	A1899 *(High affinity K_2P_3.1 inhibitor)*	Inhibition (XO)	8.1 µM (XO)	[103]
	A293 *(High affinity K_2P_3.1 inhibitor)*	Inhibition (XO)	18.1 µM (XO)	[10,15]
	Propafenone	296.1% activation at 100 µM (XO, MC)	75.4 µM (XO)	[75]
	Quinidine	57.7% activation at 100 µM (XO)	n.m.	[75]
	Mexiletine	20.6% activation at 100 µM (XO)	n.m.	[75]
	Verapamil	20.5% inhibition at 100 µM (XO)	n.m.	[75]
	Amiodarone	12.5% inhibition at 100 µM (XO)	n.m.	[75]
	Sotalol	9.8% inhibition at 100 µM (XO)	n.m.	[75]
	Ranolazine	8.3–34.88% inhibition at 100–300 µM (XO)	n.m.	[75,109]
	Barium	81.2–82.8% inhibition at 2 mM (XO)	n.m.	[67,72,73]
	Cesium	No significant effect at 1–2 mM (XO)	n.m.	[67,73]
	Arachidonic acid	No significant effect at 100 µM (XO)	n.m.	[67,73]
	Flecainide	No significant effect at 100 µM (XO)	n.m.	[84]
	Genistein	No significant effect at 100 µM (XO)	n.m.	[85]
	Carvedilol	No significant effect at 100 µM (XO)	n.m.	[75]
	Amitriptyline	No significant effect at 100 µM (XO)	n.m.	[75]
	Ajmaline	No significant effect at 100 µM (XO)	n.m.	[75]
	Vernakalant	No significant effect at 100 µM (XO)	n.m.	[83]
	Dronedarone	No significant effect at 100 µM (XO)	n.m.	[82]
	Digoxin	No significant effect (XO)	n.m.	[111]
	Digitoxin	No significant effect (XO)	n.m.	[111]
	Metoprolol	17.3% activation at 100 µM (XO)	n.m.	[75]
	Propranolol	139.2% activation at 100 µM (XO)	n.m.	[75]
	Bupivacaine	25.7% inhibition at 1 mM (XO)	n.m.	[73]
	TEA	19.9% inhibition at 1 mM (XO)	n.m.	[67]
	Quinine	17.8% inhibition at 1 mM (XO)	n.m.	[73]
	Lidocaine	13.1% inhibition at 1 mM (XO)	n.m.	[73]
	4-AP	No significant effect at 0.1–2 mM (XO)	n.m.	[67,73]
	Chloroform	44.7% inhibition at 800 µM (XO)	n.m.	[67]
	Halothane	56.4% inhibition at 800 µM (XO)	n.m.	[67]
	Isoflurane	58.4% activation at 800 µM (XO)	n.m.	[67]
*K_2P_18.1* *(TRESK)*	Vernakalant	Activation (XO, MC)	40 µM (MC)	[83]
	Isoflurane	Activation (XO)	162 µM (XO)	[61]
	Sevoflurane	Activation (XO)	224 µM (XO)	[61]
	Halothane	Activation (XO)	300 µM (XO)	[61]
	Desflurane	Activation (XO)	658 µM (XO)	[61]
	Dronedarone	29% activation at 100 µM (XO)	n.m.	[82]
	Loratadine	Inhibition (MC)	490 nM (MC)	[126]
	A1899 *(High affinity K_2P_3.1 inhibitor)*	Inhibition (XO)	900 nM (XO)	[103]
	Cloxiquine	Inhibition (MC)	3.2 µM (MC)	[130]
	Zinc	Inhibition (XO)	5–10 µM for the murine but not the human ortholog	[131]
	Arachidonic acid	43% inhibition at 20 µM (MC)	6.6 µM (MC)	[73,78]
	Lamotrigine	Inhibition (MC)	47 µM (MC)	[132]
	Bupivacaine	~75% inhibition at 100 µM (MC)	80.4 µM (XO)	[61,133]
	Tetracaine	Inhibition (XO)	496 µM (XO)	[61]
	Ropivacaine	Inhibition (XO)	610 µM (XO)	[61]
	Chlorprocaine	Inhibition (XO)	832 µM (XO)	[61]
	Mepivacaine	Inhibition (XO)	1300 µM (XO)	[61]
	Lidocaine	~70–75% inhibition at 1 mM (MC)	3.4 mM (XO)	[61,73,78]
	Mibefradil	Inhibition at 3 µM (XO)	n.m.	[131]
	Quinidine	49% inhibition at 10 µM (MC)	n.m.	[133]
	Linoleic acid	~35% inhibition at 20 µM (MC)	n.m.	[78]
	Oleatic acid	~50% inhibition at 20 µM (MC)	n.m.	[78]
	Docosahexaenoic acid	~60% inhibition at 20 µM (MC)	n.m.	[78]
	Propafenone	95% inhibition at 50 µM (MC)	n.m.	[78]
	Glyburide	76% inhibition at 50 µM (MC)	n.m.	[78]
	Quinidine	90% inhibition at 100 µM (MC)	n.m.	[78]
	Quinine	41.9–75% inhibition at 100 µM (MC)	n.m.	[61,78]
	Etomidate	30.5% inhibition at 100 µM (XO)	n.m.	[61]
	Pentobarbital	10.4% inhibition at 100 µM (XO)	n.m.	[61]
	Ketamine	14.5% inhibition at 100 µM (XO)	n.m.	[61]
	Alphaxalone	45.4% inhibition at 100 µM (XO)	n.m.	[61]
	Gabapentin	4.2% inhibition at 100 µM (XO)	n.m.	[61]
	Barium	38% inhibition at 3 mM (MC)	n.m.	[78,133]
	Ethanol	~15% inhibition at 150 mM (MC)	n.m.	[61,133]
	Apamin	No significant effect at 100 nM (XO)	n.m.	[133]
	Ruthenium red	No significant effect at 5 µM (MC)	n.m.	[133]
	Glibenclamide	No significant effect at 10 µM (MC)	n.m.	[133]
	Stearic acid	No significant effect at 20 µM (MC)	n.m.	[78]
	Digoxin	No significant effect at 100 µM (XO)	n.m.	[111]
	Digitoxin	No significant effect at 100 µM (XO)	n.m.	[111]
	Flecainide	No significant effect at 100 µM (XO)	n.m.	[84]
	Genistein	No significant effect at 100 µM (XO)	n.m.	[85]
	Tolazamide	No significant effect at 100 µM (MC)	n.m.	[78]
	Glipizide	No significant effect at 100 µM (MC)	n.m.	[78]
	Paxilline	No significant effect at 100 µM (MC)	n.m.	[78]
	Penitrem A	No significant effect at 100 µM (MC)	n.m.	[78]
	Ranolazine	No significant effect at 300 µM (XO)	n.m.	[109]
	Cesium	No significant effect at 1 mM (MC)	n.m.	[133]
	4-AP	No significant effect at 1 mM (XO)	n.m.	[73,78]
	TEA	No significant effect at 1 mM (XO) 30% inhibition at 2 mM (MC)	n.m.	[61,73,78]
	Mercury	Inhibition (XO)	n.m.	[131]
	Tetrapentyl-ammonium	Inhibition (MC)	n.m.	[130]

Potency of different drugs or compounds to activate or inhibit heterologously expressed K_2P_ currents. Compounds that are used as experimental high-affinity inhibitors of individual K_2P_ channels are highlighted in bold. Please note, however, that these compounds are by no means completely specific for single members of the K_2P_ family. IC50, mean inhibitory concentration; MC, mammalian cells; n.m., not measured; XO, Xenopus laevis oocytes.

**Table 4:** Functional evidence for K_2P_ channel expression in the cardiovascular system.

K_2P_ Channel Subunit	Species	Population/Model/Methodology	Observation	Citation
*K_2P_1.1* *(TWIK-1)*	Zebrafish	Morpholino knockdown mRNA (RT-PCR, ISH)	Knockdown of *kcnk1a* or *kcnk1b* in zebrafish embryos resulted in a phenotype atrial dilatation and bradycardia	[11]
	Mouse	CREM-transgenic murine AF model mRNA (RT-qPCR, TaqMan)	Moderate cardiac mRNA expression, V > A Ventricular mRNA downregulated in murine AF model	[16]
	Rat	Goto-Kakizaki type 2 diabetic ratsmRNA (RT-qPCR, TaqMan)	Downregulation of sinoatrial mRNA levels in Goto-Kakizaki type 2 diabetic rats	[19]
	Human	Patient-derived tissue samples mRNA (RT-PCR)	Identical mRNA levels in failing and healthy hearts	[21]
	Human	Patient-derived tissue samples	Upregulation of atrial mRNA levels in patients with atrial dilatation	[11]
	Human	Patient-derived tissue samples	Upregulation of atrial mRNA levels in patients with Brugada syndrome	[80]
	Human	Patient-derived tissue samples	Downregulation of atrial mRNA levels in AF	[12]
	Human	AF patients	Identification of three non-synonymous *KCNK1* gene variants (p.R171H, p.I98M, and p.G236S) in a cohort of 373 atrial fibrillation (AF) patients	[11]
	Human	mRNA (RT-qPCR, TaqMan)	No regulation of atrial mRNA levels in AF	[10]
*K_2P_2.1* *(TREK-2)*	Mouse	CREM-transgenic murine AF model Murine TAC model mRNA (RT-qPCR, TaqMan)	Upregulated of atrial and ventricular mRNA in a murine AF model Downregulation of atrial and ventricular mRNA in a murine TAC model	[16]
	Rat	Rat model of isoproterenol-induced left ventricular hypertrophy	Increased protein levels upon isoproterenol stimulation	[149]
	Mouse	Protein (IF)	Global K_2P_2.1 (TREK-1) knockout mice showed an exaggerated form of pressure overload-induced concentric ventricular hypertrophy, which could be prohibited only by fibroblast-specific deletion of K_2P_2.1, (TREK-1) whereas the cardiomyocyte-specific knockout of K_2P_2.1 (TREK-1) resulted in cardiac dysfunction under pressure-overload conditions	[161]
	Human	Patient-derives tissue samples mRNA (RT-qPCR, TaqMan)	Downregulation of atrial mRNA in AF	[37]
	Pig	Large animal model of burst pacing-induced AF and heart failure	Downregulation of atrial mRNA and protein Attenuation of the AF phenotype by KCNK2 gene therapy	[36,37]
	Rat	Goto-Kakizaki type 2 diabetic ratsmRNA (RT-qPCR, TaqMan)	Upregulation of sinuatrial mRNA levels in Goto-Kakizaki type 2 diabetic rats	[19]
	Human	Index patient	A heterozygous missense mutation (I267T) of K_2P_2.1 (TREK-1) was identified in a patient with idiopathic right ventricular outflow tract tachycardia	[160]
	Chicken	Isolated atrial cardiomyocytes	Resting membrane potentials of chicken embryo-derived atrial cardiomyocytes are regulated by K_2P_2.1	[153]
	Rat	Isolated rat ventricular cardiomyocytes	In isolated rat ventricular cardiomyocytes the mechano-, pH-, and arachidonic acid-sensitive potassium current *I*_KAA_ displays a number of characteristics which identify it as a K_2P_2.1 (TREK-1) current	[7,28,29,32,33,149,152]
	Mouse	*Kcnk2* knockout mouse	Phenotype of QT interval prolongation and sick sinus syndrome	[35]
*K_2P_3.1* *(TASK-1)*	Rat	Isolated rat ventricular cardiomyocytes	K_2P_3.1 (TASK-1) currents were isolated from rat ventricular cardiomyocytes by lowering pH, activation of cardiac α1-adrenergic receptors and by administration of the inhibitor A293	[15,162,163]
	Mouse	Isolated cardiomyocytes	Patch-clamp measurements of K_2P_3.1 (TASK-1) currents (controlled by knockout mice)	[45]
	Pig	Isolated atrial cardiomyocytes	Patch-clamp measurements of K_2P_3.1 (TASK-1) currents using A293: APD prolongation via K_2P_3.1 (TASK-1) inhibition	[52,53,54,164]
	Human	Isolated atrial cardiomyocytes	Patch-clamp measurements of K_2P_3.1 (TASK-1) currents using A293: APD prolongation via K_2P_3.1 (TASK-1) inhibition *I*_TASK-1_ was identified to carry up to 28% of the background potassium current in isolated human atrial cardiomyocytes	[10,39,40,53,56].
	Human	iPSC	Prolongation of APD values by transfection of K_2P_3.1 (TASK-1) siRNA	[22]
	Zebrafish	Morpholino knockdown	Decreased heart rate was observed after K_2P_3.1 (TASK-1) knockdown	[165].
	Mouse	CREM-transgenic murine AF model Murine TAC model mRNA (RT-qPCR, TaqMan) and protein (WB)	Downregulation of atrial mRNA and protein level in murine AF model Downregulation of atrial mRNA and protein level in murine TAC model	[16]
	Guinea pig	Excised guinea pig hearts	Prolongation of atrial effective refractory periods upon TASK-1 inhibition at pH 7.8	[49]
	Mouse	*Kcnk3* knockout mouse	Phenotype of QTc prolongation (around 30%), prolongation of single cell APDs or monophasic action potentials and a broad QRS complex	[47]
	Rat	*Kcnk3* knockout rat	Phenotype of cardiomyocyte APD prolongation as well as resting membrane depolarization	[15]
	Dog	Dog model of postoperative AF	Downregulation of atrial TASK-1 expression in postoperative AF	[50]
	Pig	Large animal model of burst pacing-induced AF	Upregulation of atrial TASK-1 expression and currents Acute cardioversion upon TASK-1 inhibition Rhythm control of AF upon TASK-1 gene therapy of pharmacological TASK-1 inhibition	[52,141,164]
	Human	mRNA (RT-qPCR, TaqMan), protein (WB)	Upregulation of atrial TASK-1 expression and currents in cAF	[10,41,55,57]
	Human	AF patient cohort	Three genetic *KCNK3* variants which reduce the expression or channel function were found in patients with familial AF	[49]
	Mouse	*Kcnk3* knockout mouse	Compared to wild-type littermates, *Kcnk3* knockout mice showed a preservation of systolic as well as diastolic function and a relative abrogation in concentric left ventricular hypertrophy upon TAC-induced pressure overload	[46]
	Human	Patient cohorts	*KCNK3* loss-of-function mutations were found to cause idiopathic pulmonary arterial hypertension	[166]
	Human	Patient-derived tissue samples mRNA (RT-qPCR, TaqMan) and protein (WB)	Upregulation of atrial mRNA and protein in AF Downregulation of atrial mRNA in heart failure	[40]
*K_2P_4.1* *(TRAAK)*	Mouse	*Kcnk4* knockout mice	No obvious cardiac phenotype reported	[167,168]
	Human	Patient-derived tissue samples mRNA (RT-qPCR)	Downregulation of ventricular mRNA levels in non-ischemic heart failure	[22]
	Human	Patient-derived tissue samples mRNA (RT-qPCR, TaqMan)	No regulation of atrial mRNA levels in AF patients	[10]
*K_2P_5.1* *(TASK-2)*	Mouse	*Kcnk5* knockout mice	Observation of subviable phenotype and sudden unexplained dead but association with arrhythmia or cardiomyopathy remains speculative as no detailed cardiac characterization was reported	[169]
	Mouse	CREM-transgenic murine AF model mRNA (RT-qPCR, TaqMan)	No regulation of atrial mRNA in murine AF model	[16]
	Rat	Goto-Kakizaki type 2 diabetic rats mRNA (RT-qPCR, TaqMan)	Downregulation of sinoatrial mRNA levels in Goto-Kakizaki type 2 diabetic rats	[19]
	Human	mRNA (RT-qPCR, TaqMan)	Trend towards downregulation of atrial mRNA levels in AF	[10]
*K_2P_6.1* *(TWIK-2)*			*Physiological role under debate because of low currents upon recombinant expression*	
	Mouse	CREM-transgenic murine AF model Murine TAC model mRNA (RT-qPCR, TaqMan)	No regulation in murine AF model Upregulation of atrial mRNA in murine TAC model	[16]
	Rat	Goto-Kakizaki type 2 diabetic rats mRNA (RT-qPCR, TaqMan)	Downregulation of sinoatrial mRNA levels in Goto-Kakizaki type 2 diabetic rats	[19]
	Mouse	*Kcnk6 knockout mouse*	*Kcnk6* knockout mice are hypertensive and display elevated RV pressure level as well as enhanced vascular contractility	[170,171,172]
	Human	Patient-derived tissue samples mRNA (RT-qPCR, TaqMan)	No regulation of atrial mRNA in AF patients	[10]
*K_2P_7.1* *(TWIK-3)*	Human, Mouse	mRNA (RT-qPCR, TaqMan)	*Most studies show* *very low cardiac mRNA levels. Functionality of the channel still under debate.*	[16] al. 2015) Wang et al. 2018)
	Human	Patient-derived tissue samples mRNA (RT-qPCR)	Upregulation of atrial mRNA levels in AF	[63]
	Human	Patient-derived tissue samples mRNA (RT-qPCR, TaqMan)	No mRNA regulation in AF	[10]
	Mouse	*Kcnk7* knockout mouse	No cardiac phenotype of the *Kcnk7* knockout mouse has been described	[173]
*K_2P_9.1* *(TASK-3)*	Human	Genetic disease	*KCNK9* imprinting syndrome linked to obstructive sleep apnea	
	Human	Patient-derived tissue samples mRNA (RT-qPCR)	Downregulation of ventricular mRNA levels in heart failure	[22]
	Human	Patient-derived tissue samples mRNA (RT-qPCR, TaqMan)	Trend towards upregulation in AF	[10]
	Mouse	*Kcnk9* knockout mouse	Phenotype of concentric left ventricular hypertrophy with preserved ejection fraction	[46]
	Human	Single channel patch-clamp measurements on isolated human atrial cardiomyocytes	Evidence for heteromeric K_2P_9.1/ K_2P_3.1 but not for K_2P_9.1 homodimers	[56]
*K_2P_10.1* *(TREK-2)*	Human, mouse	Patient-derived tissue samples, CREM-transgenic murine AF model, Murine TAC model, mRNA (RT-qPCR, TaqMan)	No regulation of atrial mRNA levels in AF patientsNo regulation of atrial or ventricular mRNA levels in a murine AF modelNo changes in ventricular mRNA levels in a murine TAC modelUpregulation of left and right atrial mRNA in heart failure patients	[41]
	Mouse	*Kcnk10* knockout mouse	No cardiac phenotype of the *Kcnk10* knockout mouse has been described	[174]
	Human	Patient-derived tissue samples mRNA (RT-qPCR, TaqMan)	No regulation of atrial mRNA levels in AF patients	[10]
*K_2P_12.1* *(THIK-2)*	Human, Rat, Mouse	mRNA (NB, RT-PCR, RT-qPCR, TaqMan)	*Most studies show* *very low cardiac mRNA levels. Functionality of the channel still under debate.*	[10,15,16,66,67]
*K_2P_13.1* *(THIK-1)*	Human	Patient-derived tissue samples mRNA (RT-qPCR, TaqMan)	Downregulation of atrial mRNA level in cAF patients	[10]
	Human	Patient-derived tissue samples mRNA (RT-qPCR, TaqMan)	Trend towards downregulation of atrial mRNA level in heart failure patients	[40]
	Pig	Large animal model of burst-pacing induced AF and heart failure	Downregulation of atrial protein expression in combined AF and heart failure	[129]
*K_2P_15.1* *(TASK-5)*	Human, Rat, Mouse	mRNA (RT-PCR, RT-qPCR)	*Most studies show* *rather low cardiac mRNA levels. Functionality of the channel still under debate.*	[10,15,69,70] Wiedmann et al. 2018)
	Human	Patient-derived tissue samples mRNA (RT-qPCR, TaqMan)	No regulation of atrial mRNA levels in cAF patients	[10]
	Mouse	CREM-transgenic murine AF model mRNA (RT-qPCR, TaqMan)	Downregulation of atrial mRNA levels in murine AF model	[16]
*K_2P_16.1* *(TALK-1)*	Human, Rat	mRNA (NB, RT-PCR, RT-qPCR, TaqMan)	*Most studies show* *negligible or low cardiac mRNA levels*	[10,15,60,67,71]
*K_2P_17.1* *(TALK-2)*	Human	Patient-derived tissue samples, iPSC mRNA (RT-qPCR)	Downregulation of ventricular mRNA levels in non-ischemic heart failure iPSC: *KCNK17* knockdown led to APD prolongation	[22]
	Human, Mouse	Index patient HL-1 cells (cultured cardiomyocyte cell line), mRNA (RT-qPCR)	A patient suffering from progressive and severe cardiac conduction disorder in combination with idiopathic ventricular fibrillation was identified to carry both, a splice site mutation in the sodium channel gene *SCN5A* as well as a gain-of-function mutation in the *KCNK17* gene HL-1 cells: KCNK17 *knockdown* overexpression led to APD shortening	[5]
	Human	Index family Patient derived iPSC	A common *KCNK17* gain-of-function variant might be protective for LQTS by promoting APD shortening	[74]
	Human	Patient-derived tissue samples, mRNA (RT-qPCR, TaqMan)	Downregulation of right atrial mRNA levels in cAF	[10]
	Human	Patient-derived tissue samples, mRNA (RT-qPCR, TaqMan) and protein (WB)	Downregulation of left and right atrial protein and mRNA level in HF	[40]
*K_2P_18.1* *(TRESK)*	Zebrafish, Mouse, Human	mRNA (ISH, RT-PCR, RT-qPCR, TaqMan)	*Most studies show* *negligible cardiac mRNA levels*	[10,16,61,76,77,78]

Evidence in literature for cardiac relevance of K_2P_ channel subunits. A, expression in atrial tissue; AF, atrial fibrillation; HF, heart failure; IF, immunofluorescence; iPS, induced pluripotent stem cell; ISH, in situ hybridization; LA, left atrium; NB, Northern blot; RT-PCR, reverse transcriptase PCR; RT-qPCR, reverse transcriptase quantitative PCR; RA, right atrium; TAC, transverse aortic constriction; TaqMan, reverse transcriptase quantitative PCR employing TaqMan^®^ hydrolyse probes to increase specificity; V, expression in ventricular tissue; WB, Western blot.
